# Supplementary figures and images for: Non-genetic differences underlie variability in proliferation among esophageal epithelial clones
Source: PLoS Comput Biol. 2024 Oct 28;20(10):e1012360. doi: 10.1371/journal.pcbi.1012360 (PMC11573201; doi:10.1371/journal.pcbi.1012360)

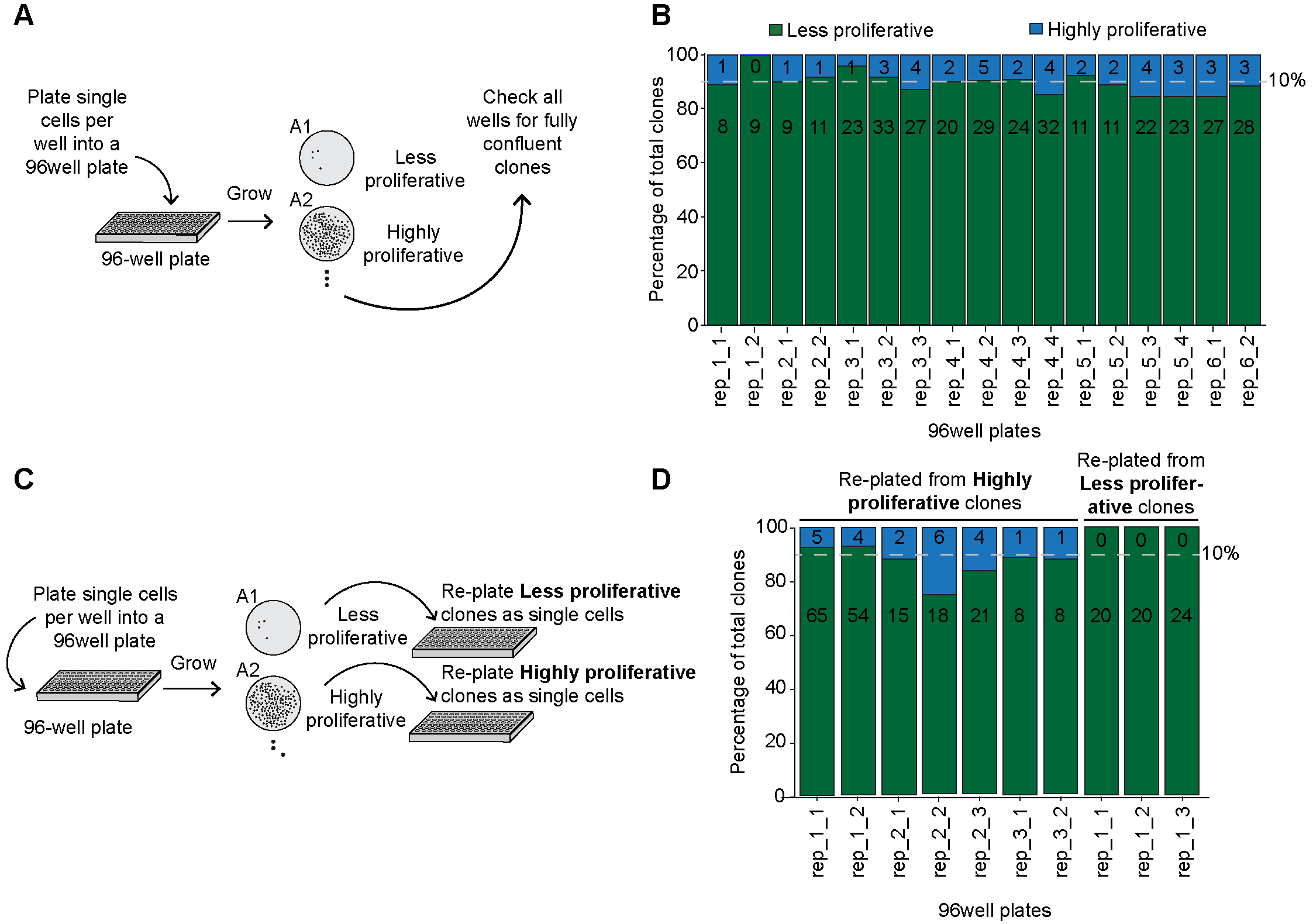

Supplement: S1 Fig — (A) Experimental design for clonal isolation and characterization. Single EPC2-hTERT cells were plated in individual wells of a 96-well plate, and their growth was monitored over 14 days. Clones filling or nearly filling the well within this period were classified as highly proliferative, while those that did not were labeled as less proliferative. (B) Stacked bar plot of ratios depicts the proportion of highly proliferative and less proliferative clones (n = 6 biological replicates). (C) Schematic of replating experiment following the initial characterization, clones identified as highly proliferative or less proliferative were dissociated into single cells and replated into new 96-well plates. These replated cells were then allowed to grow for another 14-day period to reassess their proliferative status. (D) Bar plot of the ratio between highly proliferative and less proliferative subclones as described in S1C Fig (n = 3 biological replicates). (TIF) [file pcbi.1012360.s001.tif]

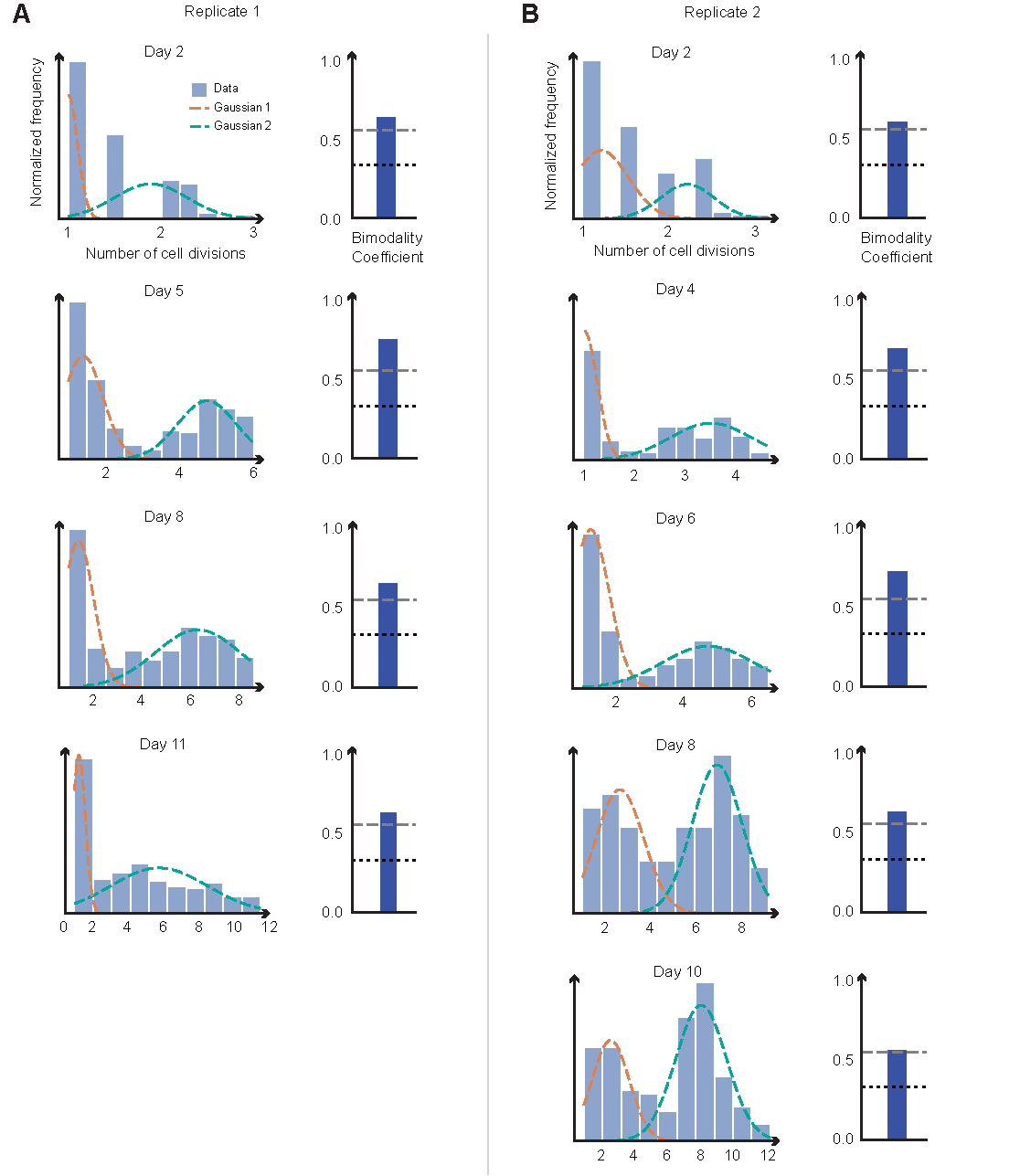

Supplement: S2 Fig — (A-B) Histograms showing the number of cell divisions for each clone based on the number of cells observed. Each set column corresponds to a separate biological replicate. Histograms are fitted with two Gaussians using a Gaussian Mixture Model (GMM). Each histogram is paired with a bar plot on the right, depicting the corresponding Bimodality Coefficient. The dashed gray lines indicate the 5/9 threshold (suggestive of a likely bimodal distribution) and the dashed black lines indicate the 1/3 threshold (suggestive of a unimodal distribution). (TIF) [file pcbi.1012360.s002.tif]

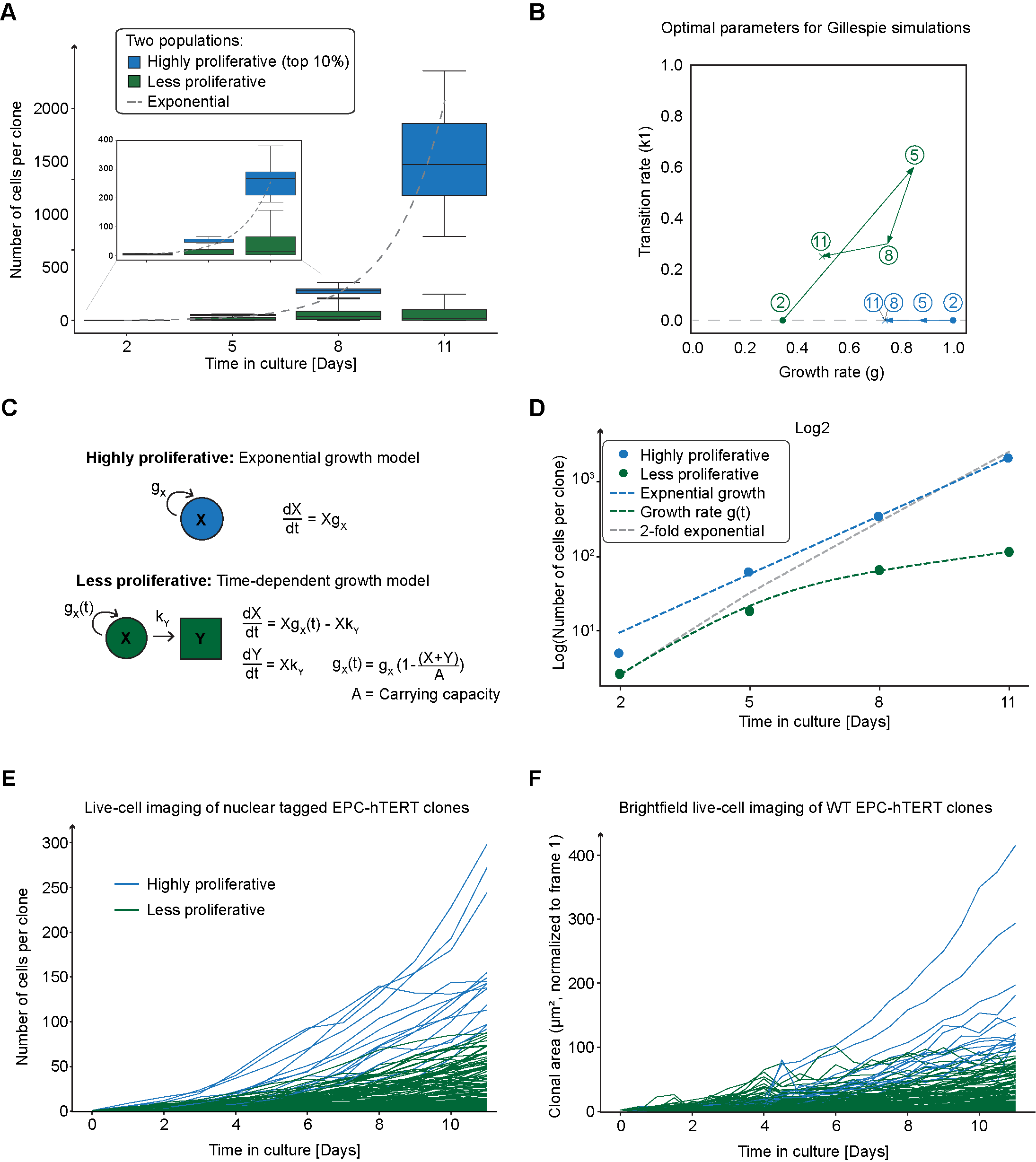

Supplement: S3 Fig — (A) Bar plot showing cell divisions per clone over time (data from Fig 1C), with highly proliferative (blue) and less proliferative (green) cells. A solid gray line highlights exponential growth for comparison. (B) Scatter plot of the optimal parameters for the Gillespie simulations for each time point, the time point is labeled at each point. The parameters for highly proliferative and less proliferative clones are in blue and green respectively. (C) Schematic of growth models for the highly proliferative and less proliferative clones, with highly proliferative clones fitted using an exponential growth model and less proliferative clones requiring a logistic growth model for accurate data representation. (D) Scatter plot of highly proliferative and less proliferative data fitted by their respective growth rate models shown in panel C. (E) Live-cell imaging data from mCherry-labeled EPC2-hTERT cells over 11 days. Line plot shows the number of cells per clone as measured at 24-hour intervals. Highly proliferative clones (the top 10% largest clones) are depicted in blue, while less proliferative cells are shown in green (n = 2 technical replicates, combined). (F) Live-cell imaging data from phase imaging of unlabeled EPC2-hTERT cells over 11 days. Line plots of normalized clonal area (μm2) over time, measured at 12-hour intervals, for EPC2-hTERT cells (n = 2 technical replicates, combined). (TIF) [file pcbi.1012360.s003.tif]

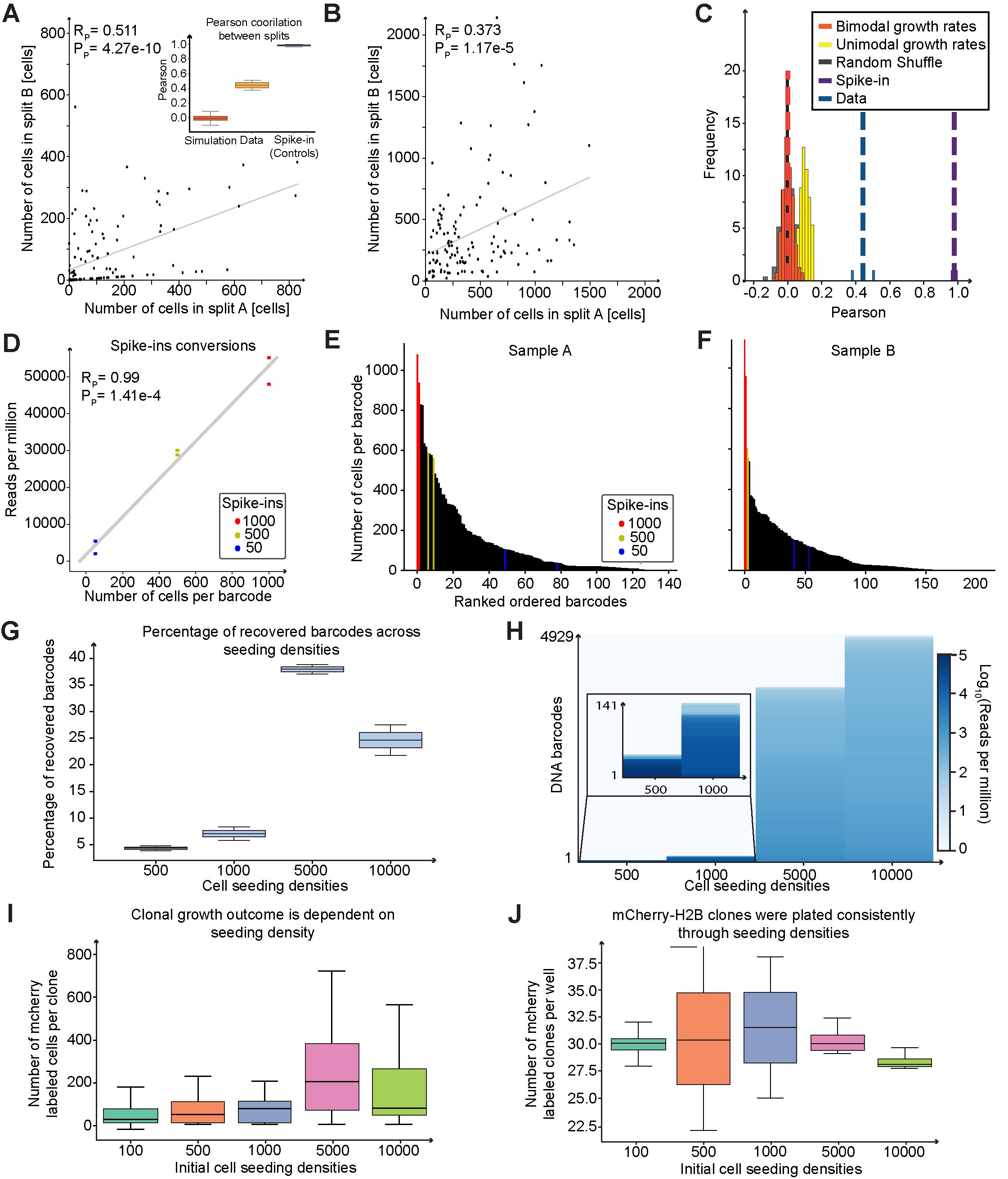

Supplement: S4 Fig — (A-B) Scatter plot depicting the number of cells in sample A and B for each barcode captured by sequencing. The Pearson correlations were 0.511 and 0.373 with p-values of 4.27e-10 and 1.17e-5 in the two replicates shown in A and B. The embedded box plots summarize the findings comparing simulated data, our experimental replicates, and spike-in positive controls that should show high correlation (n = 2 technical replicates). (C) Histograms showing the Pearson correlations for simulated data (see methods section: Heritability analysis), experimental data, and spike-ins positive controls. Both replicates, shown in blue, fall outside the simulation ranges, indicating memory of epithelial cell proliferative capacity. (D) Scatter plot showing data from spike-ins included in the experiment with the number of cells versus the number of reads per million for the barcode captured by sequencing. (E-F) Bar plots of rank-ordered barcodes, representing barcode distribution after 8 days of growth. (G) Box plot of percentage of recovered barcoded cells at 500,1000, 5000 and 10000 cells per well, detailed in methods “Clonal density experiments” (n = 2 technical replicates). (H) Rank-ordered barcodes grouped by density while the heat bar displays the log(Reads per Million). (I) Box plot of data from Fig 3D showing the number of mCherry labeled cells per clone with the data split by initial seeding density (n = 2 biological replicates each with 6 technical replicates). (J) Box plot showing quantification of the initial seeding density for the experiment in Fig 3D to confirm that each condition started with the same number of mCherry labeled cells. The plot depicts the number of mCherry clones measured at day 8. (TIF) [file pcbi.1012360.s004.tif]

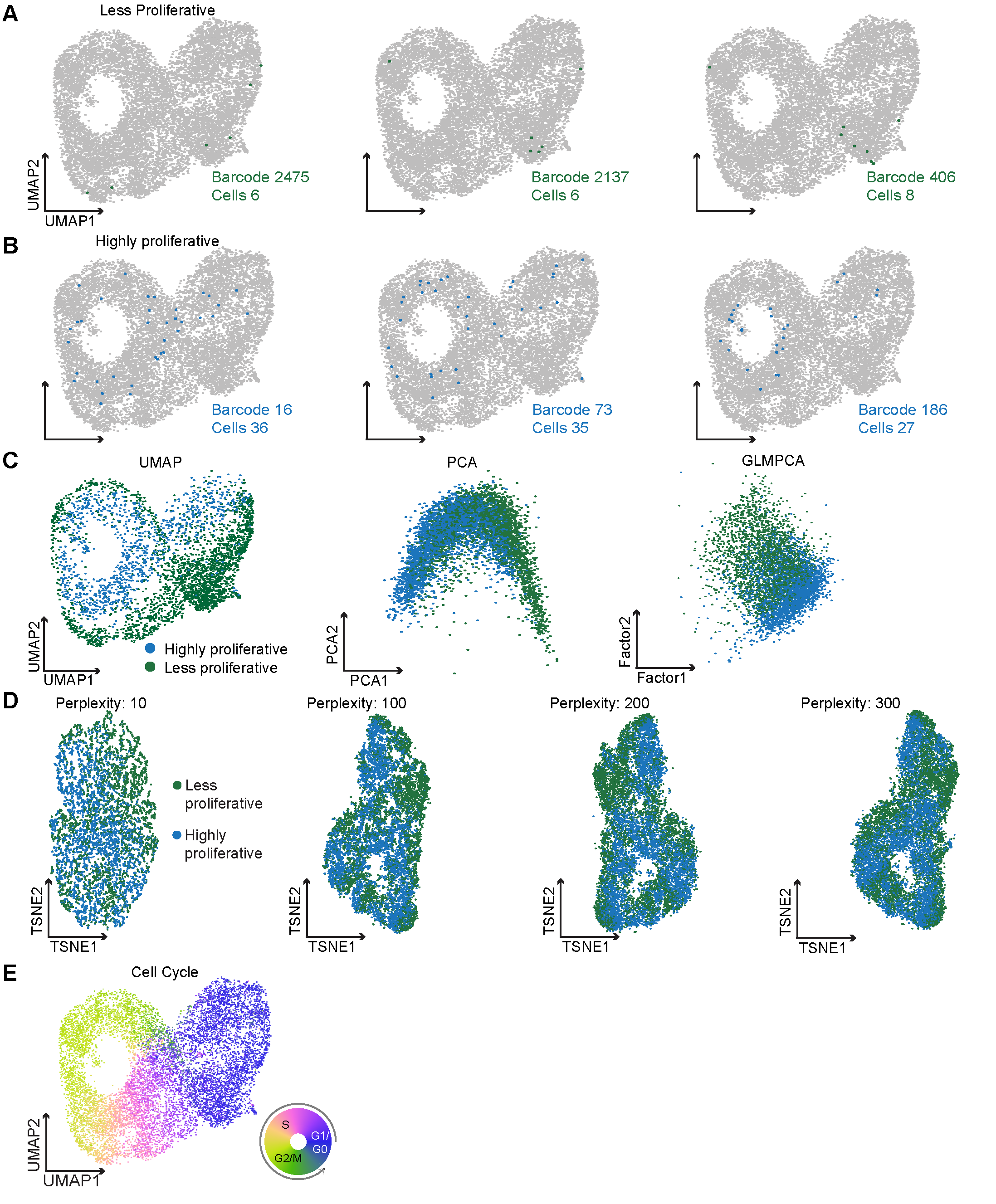

Supplement: S5 Fig — (A) UMAP plots with examples of lineages from less proliferative clones. (B) UMAP plots with examples of lineages from highly proliferative clones. (C-D) Dimensionality reduction plots of scRNA-seq data. These plots utilize different dimensionality reduction methods (including UMAP, PCA, GLMPCA, and t-SNE with varying perplexity settings of 10, 100, 200, 300, and 400) to visualize scRNA-seq data. Cells are color-coded to distinguish between highly proliferative and less proliferative states, which we assigned based on barcodes. (E) UMAP projection of scRNA-seq data with cells colored based on their cell-cycle position using a circular color scale. Discrete stage labels are placed in approximate positions on the circular legend. (TIF) [file pcbi.1012360.s005.tif]

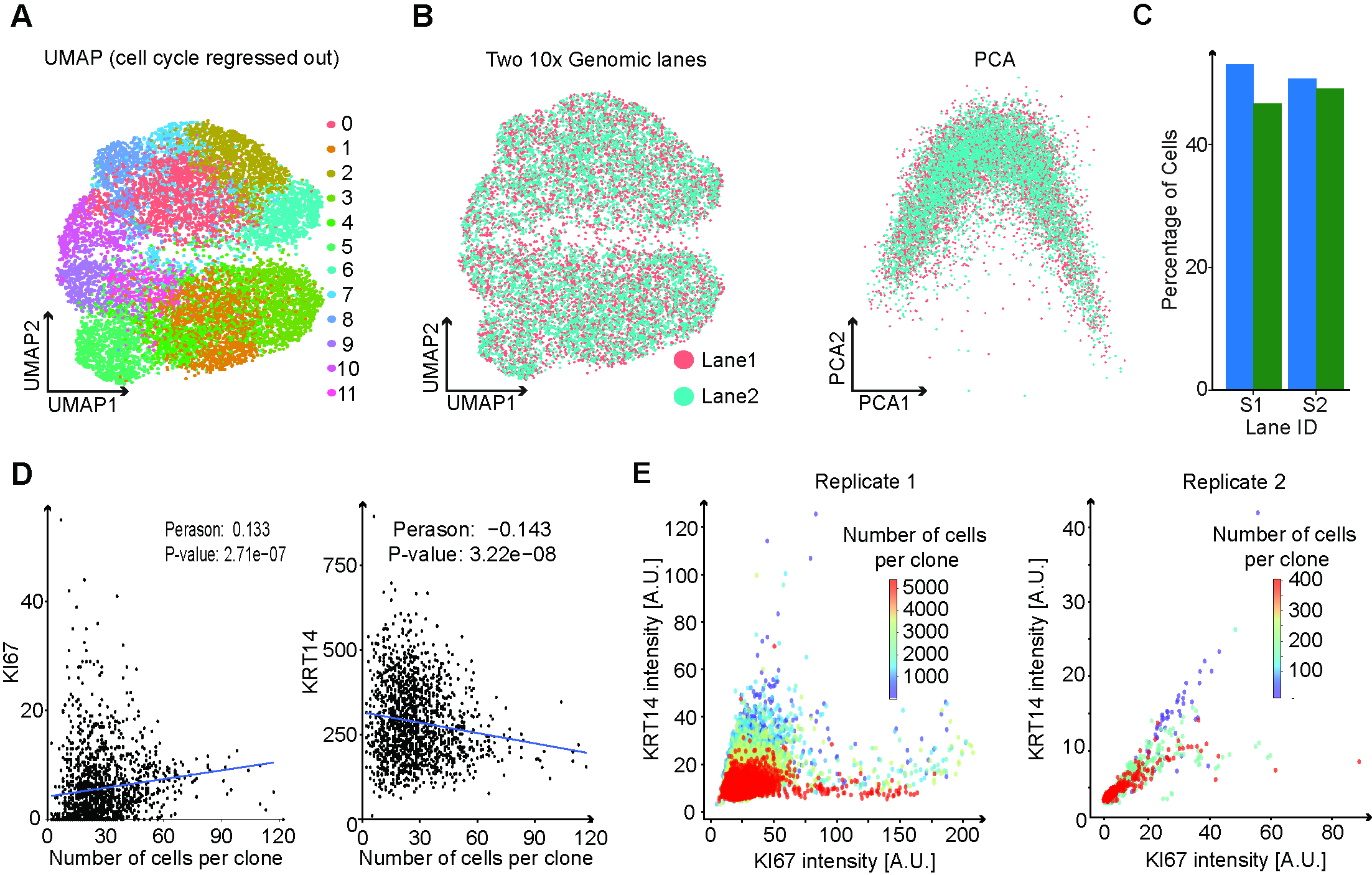

Supplement: S6 Fig — (A) UMAP plot with regressed out of cell cycle Influence, cells are labeled according to Louvain clusters. (B) UMAP and PCA plots of cells processed through two different 10x Genomics lanes illustrate the consistency and distribution of the cells across the lanes. (C) Barplot showing the percentage of cells in each state split by lane 1 and lane 2. (D) Scatter plot of the number of cells per clone against MKI67 (left) and KRT14 (right) expression from the EPC2-hTERT scRNA-seq dataset. (E) Immunofluorescence data in Fig 5 staining for Ki67 and KRT14 in clones of different sizes. Scatter plot shows Ki-67 and KRT14 intensities with each cell labeled according to the total number of cells in its respective clone (n = 2 biological replicate and n = 2 technical replicates). (TIF) [file pcbi.1012360.s006.tif]

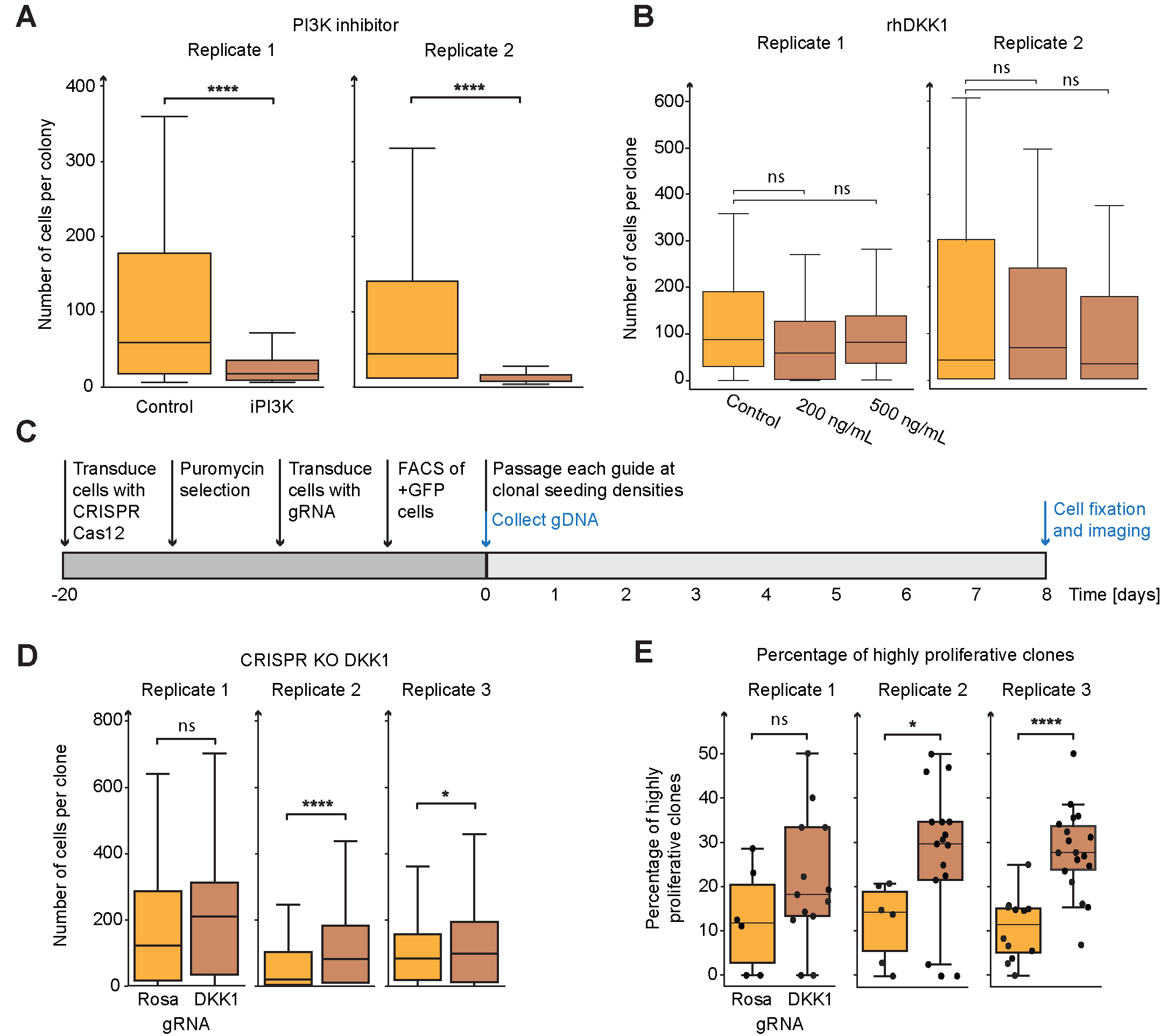

Supplement: S7 Fig — (A) Experiment testing the effects of PI3K inhibitor on the growth of clones over 8 days. Box plot shows the number of cells per clone with and without PI3K inhibitor (iPI3K) (n = 2 biological replicates and n = 2 technical replicates) (p-value equal to 4.33e-5 and 1.08e-9 respectively). (B) Experiment testing the effects of recombinant DKK1 protein on growth of clones over 8 days. Box plot shows the number of cells per clone at two different concentrations of human recombinant DKK1 (n = 2 biological replicates and n = 3 technical replicates per dosage). (C) Outline of experimental design for the CRISPR-Cas12a targeted knockout (KO) used to investigate the role of DKK1 in clone expansion. (D) Box plot showing the number of cells per clone split by gRNA targeting Rosa26 and DKK1 (n = 3 biological replicates) (p-value equal to 1.81e-1, 5.54e-12 and 1.06e-2 respectively). (E) Box plot showing the percentage of highly proliferative clones per well split by gRNA targeting Rosa26 and DKK1 (n = 3 biological replicates each with 6 technical replicates) (p-value equal to 2.17e-01, 4.16e-02 and 6.90e-05 respectively). We selected cutoff size for a highly proliferative clone by taking the top 10% of the average of the Rosa26 control data. We then used that cutoff size to determine which clones are highly proliferative in the DKK1 KO data. (TIF) [file pcbi.1012360.s007.tif]

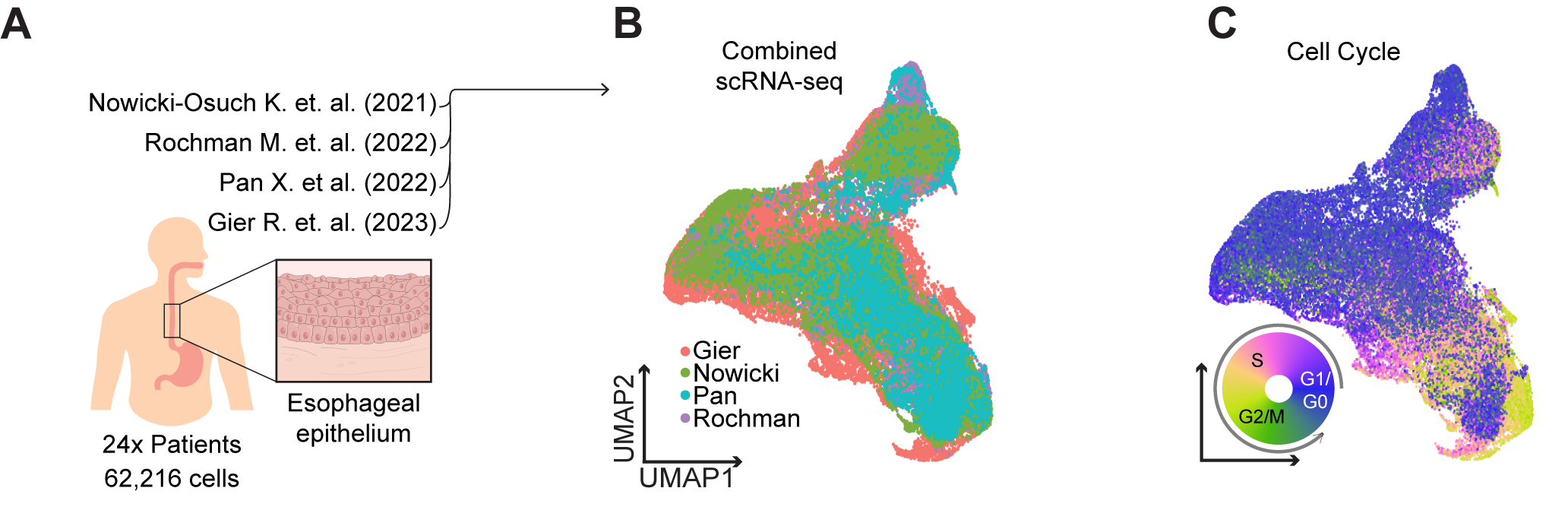

Supplement: S8 Fig — (A) A schematic of the integration of numerous published scRNA-seq datasets for healthy esophageal epithelium comprising 24 patients and 62,216 cells. (B) UMAP plot showing each cell labeled by its dataset after batch correction. (C) UMAP plot in which the color of each cell shows their predicted cell-cycle position using a circular color scale. Discrete stage labels are placed in approximate positions on the circular legend. (TIF) [file pcbi.1012360.s008.tif]
