# Supplementary material for: Non-genetic differences underlie variability in proliferation among esophageal epithelial clones
Source: PLoS Comput Biol. 2024 Oct 28;20(10):e1012360. doi: 10.1371/journal.pcbi.1012360 (PMC11573201; doi:10.1371/journal.pcbi.1012360)
Supplement: Attachment — Submitted filename: PCB_Response_to_Reviewers (1).pdf [file pcbi.1012360.s012.pdf]

Point-by-point response to reviews for PLOS Comp Bio

Responses text is in blue

Reviewer #1:

The manuscript, “Non-genetic differences underlie variability in proliferation among esophageal epithelial clones”, describes a phenomenon where proliferation capacity of clones are inherited over cell passages through non-genetic means. This is not the first study of its kind but it brings some novel approaches such as barcoding into the mix. The authors present some elegant ways to decipher between multiple hypotheses. There are some good validation experiments performed perturbing certain signaling pathway proteins. However, we are not sure why this is in PLOS Comp Bio as it has very little computational biology presented (only simple 1 or 2 states models presented). Overall we think it is an interesting study.

> Thank you, Reviewer #1, for the thorough and constructive feedback on our manuscript. We have carefully considered each of your comments and suggestions and have made significant improvements to the manuscript, both in content and clarity. We have also moved the modeling into the main figure of the paper and discussed the biological implications of the model.

Major comment

• Make sure all panels in all figures should be mentioned in the main text.

> We thank the reviewer for pointing this out. All figures and their respective panels have now been correctly mentioned in order in the main text.

• The tables mentioned in text cannot be found.

> The missing tables have now been added to the supplementary materials.

• The methods are very general and hard to follow from the main text. Please make sure methods are detailed enough to enable readers to repeat the experiments and simulations.

> We recognize the need for clearer methods and have expanded this section to provide greater detail, ensuring readability and reproducibility.

• Sample size, both number of biological and of technical replicates, need to be reported.

> We thank the reviewer for pointing this out. We have explicitly reported the number of biological and technical replicates of each experiment in the captions for each figure and in the methods section of the manuscript.

• Figure1: Individual epithelial clones have unique capacities for growth and differentiation

o The authors used top 10% of clones ranked by size as a criterion to assign whether a clone is expanding or committed. However, it is unclear how this threshold was selected. The authors

did not mention other criteria thresholding in other sections of the paper, but there is at least one place where the percentage of expanding colonies is changed (Fig S4C), which differs from the definition of expanding clones as the top 10% in size and suggests that the authors used other thresholds in that experiment.

We thank the reviewer for directing our attention to the need to better define this threshold. We selected the top 10% as highly proliferative based upon the experiments in Fig. S1. Specifically, we plated single cells into wells of 96-well plates and measured the fraction of clones that grow until they fill or nearly fill an entire well.

The only place in the manuscript where we adjust this threshold is in the context of perturbations, where we expect a shift in the number of highly proliferative clones. Thus, in the experiment in Fig S4C (now Fig. S6G), we used the 10% threshold to identify the highly proliferative clones in the control sample (with the Ros26 gRNA). From the control, we determined the minimum number of cells per clone in the highly proliferative clones. We then use this cutoff to determine how many clones of the gRNA DKK1 KO are above the threshold. We have updated the text to make sure this is clearly described for Fig. S4C (lines 302-305 and 1181-1183). This is the only place in the manuscript where we used any methodology other than assuming the highly proliferative clones are the top 10%. We needed to alter the threshold as we were observing more of the highly proliferative clones in the DKK knockout sample. Here, we defined highly proliferative clones for Rosa and DKK1 gRNA based on the number of cells per clone required for the average Rosa26 replicate to contain 10% such clones. We also show this same data in Fig S4B (now Fig. S6F), but with boxplots of the total number of cells per clone in the Rosa control and DKK knockout.

o The authors assumed that each clone from a cell line is genetically identical. This is probably not the case as cell lines can generate genetic variation very quickly in a few generations. The genetic similarity amongst clones can be tested by small sample bulk sequencing.

We thank the reviewer for this point and fully agree that cell lines, over generations, have the potential to accumulate genetic changes.

However, we experimentally showed that the highly proliferative clones are not permanently in the proliferative state, which would be expected if they harbored a genetic mutation that enabled their growth. In Fig. S1C,D, we isolated the proliferative clones from individual wells of a 96-well plate and then split them up into single cells again. If a mutation were enabling proliferation, we would expect that most, if not all, cells would be proliferative. Rather, we find that upon redistribution into individual wells, these clones show a similar distribution of proliferation states to the initial population. This experiment led us to conclude that the proliferation phenotype is reversible, and thus is likely not due to an irreversible genetic difference between these cells. We recognize that genetic changes do likely accumulate in these cells through time (as they

do with most cell lines), but this experiment allows us to conclude that the proliferative phenotype is able to reverse.

To further address this concern, we also explored whether copy number differences correlate with the proliferative phenotype. To assess this possibility, we analyzed our scRNA-seq data in Fig 4D (now Fig. S4C) using inferCNV (version: infercnv_1.14.2), an established tool for inferring copy number alterations. In this dataset, we have assigned the proliferative phenotype to each cell (based upon the barcodes, Fig. S4A,B) and then we asked whether the proliferative cells have copy number variations (CNVs) that are not present in the less proliferative cells. We defined the reference to be the less proliferative clones and observed any potential aberrations on the highly proliferative clones. We used the default (0.1) cutoff parameter for 10x Genomics to distinguish between low and high expression. We also used a Hidden Markov Model (HMM) to convert the smoothed gene expression data into genomic regions likely affected by CNVs.

From this analysis, we did not observe copy number alterations specific to the less proliferative or highly proliferative clones (Reviewer Figure 1). Broadly, we observe minimal copy number alterations, indicating a relative genetic stability and similarity among these cells. Together, the absence of distinct copy number change in the highly proliferative or less proliferative clones further reinforces our claim that these proliferative differences are not derived from genetic differences in the cells.

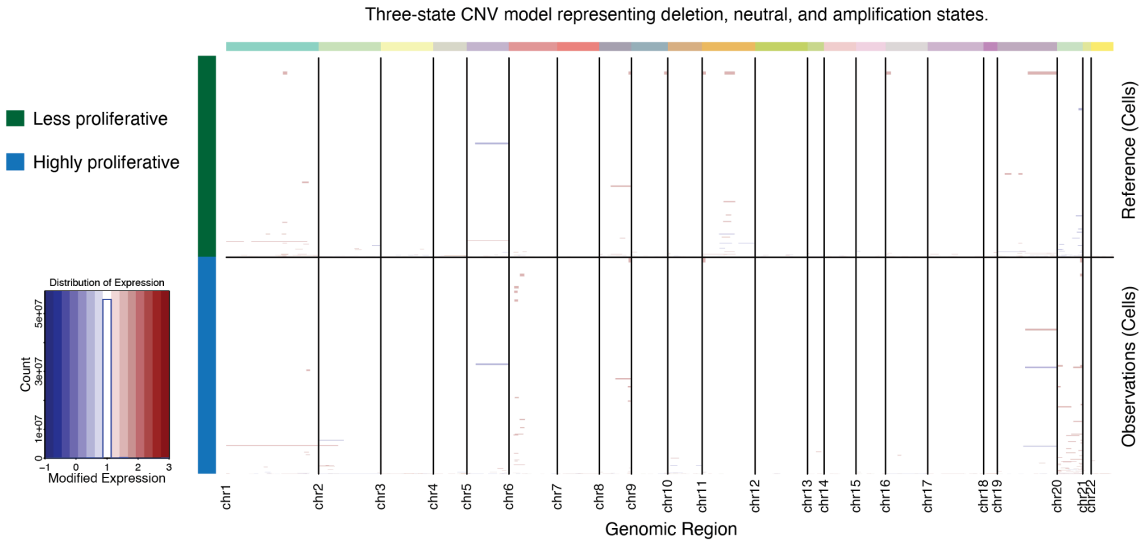

Reviewer Figure 1: Highly proliferative and less proliferative clones have similar copy number profiles predicted from scRNA-seq.

o Fig S1A: the author should also test if cells picked from the committed clone can also give rise to both committed and expanding clones. It was only tested that the expanding clone can give rise to the committed.

We agree with this reviewer that it would be informative to test whether the less proliferative clone can give rise to both types of clones. We tried these experiments by isolating these less proliferative clones from individual wells of 96-well plates and then plating them again at single cell density. We found that these single cell clones (derived from the less proliferative clones) did not grow over 14 days. One challenge, however, is that we do not know if we had waited longer or tested more of these less proliferative clones if we would see the proliferative phenotype appear. Given this result, we have updated the text now to clearly specify that we did not directly observe the less proliferative clones giving rise to highly proliferative clones. While it's possible that such a transition from less to highly proliferative states could happen, it appears to be a rare event. This rarity, coupled with the typically small number of cells in less proliferative clones, likely led to this phenomenon not being captured in our experiments. This data is now described in lines 133-134, and shown in supplemental figure Fig S1C,D.

• Figure 2: Cell states underlying proliferative potential are heritable through cell division
o Fig 2B and S2A: even though the p-values are low, the correlation coefficients of 0.511 and 0.373 should not be considered high, and the data points seem to have high variation. The 4 axes from the two plots show very different range of cell numbers. Again, the number of experimental replicates is not stated clearly in main text, and a reason for plotting the results in separate plots are not clarified. It is not convincing enough to draw the conclusion that the proliferation capacity between splits A and B are correlated.

We agree with the reviewer that these are not very high correlation coefficients. However, here we are testing whether these correlations are higher than would be observed with a random distribution of growth states. We acknowledge that this wording could be more precise, and we have updated the manuscript accordingly (lines 183-192). To clarify this data and the interpretation, we have updated the analysis in the main figure. Rather than looking at the correlation, we took the top 10% of clones in each sample and compared them. We used transcripts per million of each barcode as a proxy for size. We then calculated and visualized the overlap between the clones from each sample.

The data is represented as Venn diagrams, which provide a clear and intuitive depiction of the extent of overlap in highly proliferative clones between the samples. We also performed simulations, where each cell was assigned a random growth rate. This model represents a null hypothesis, assuming no memory of prior proliferative states in sibling cells. Comparing the overlap of highly proliferative clones between the experimental replicates and the simulation, we found a consistently higher overlap in the actual data. This result suggests that the proliferative capacity of these epithelial cells is not random, but has a heritable component.

The number of experimental replicates have been added to the figure legend and method section.

o Furthermore, since it has already been shown the impact of cell density in the next section, there may be variation in cell density in the two split plates. Have the authors consider growing the individual split cells in separate wells? This way, the density variable can be removed (actually we are not sure why this is not the experimental design to begin with as it is a more straight forward experiment)

Our study uses both of these experimental designs proposed by the reviewer, the 96-well plate and sparsely plated 6-well plate. We use 96-well plates, each well containing a single cell, to establish the phenomenon (Fig. S1A,B) and to facilitate replating of individual clones after observing their growth (Fig. S1C,D). For experiments aimed at quantifying the growth of many clones, sparse plating in a 6-well format simplifies imaging (particularly at large numbers such as in Fig. 1B,C). We can also more effectively recover the small clones when using 6-well plates as is done in our barcoding experiment in Fig. 2A,B (now Fig. 3A,B) and scRNA-seq experiment in Fig. 3A-D (now Fig. 4A-E). Therefore, our experimental design choice depended on the specific goal and downstream profiling or analyses and the main conclusions of the study hold across the different experimental designs.

o A time period of 4 doublings is used. Can the authors explain the choice of this interval. Will the correlation change if the interval varies?

The choice of a 4-day doubling period was selected to balance two factors. First, we wanted to allow for enough doublings such that both samples (split from the same initial pool) would be likely to contain nearly all barcodes. Second, we wanted to minimize the total number of doublings as we expect that cells with the same barcode will be less like each other as they proliferate more. Thus, four doublings was the minimum where we would still have each barcode likely represented across the two samples.

We have included a more detailed explanation of these considerations in the revised manuscript (lines 181-184).

• Figure 3: The transcriptional state of expanding and committed cells are distinct
o Fig 3D: no clear clusters are formed between the two states. Although the expanding cells locate more in the inner circle on the UMAP, that may be due to batch effects. The authors have not mentioned the number of batches/replicates, and the experiment should be repeated.

We have added more experimental information to the manuscript for Figure 3 (now Fig. 4), lines 245-249. Our experiment contained two separate lanes of 10X scRNA-seq, and thus we have now added Fig. S5B to show the different lanes and how the data integrates. Broadly, we do not see strong batch effects and the batch differences do not correlate with the proliferative states Fig. S5C.

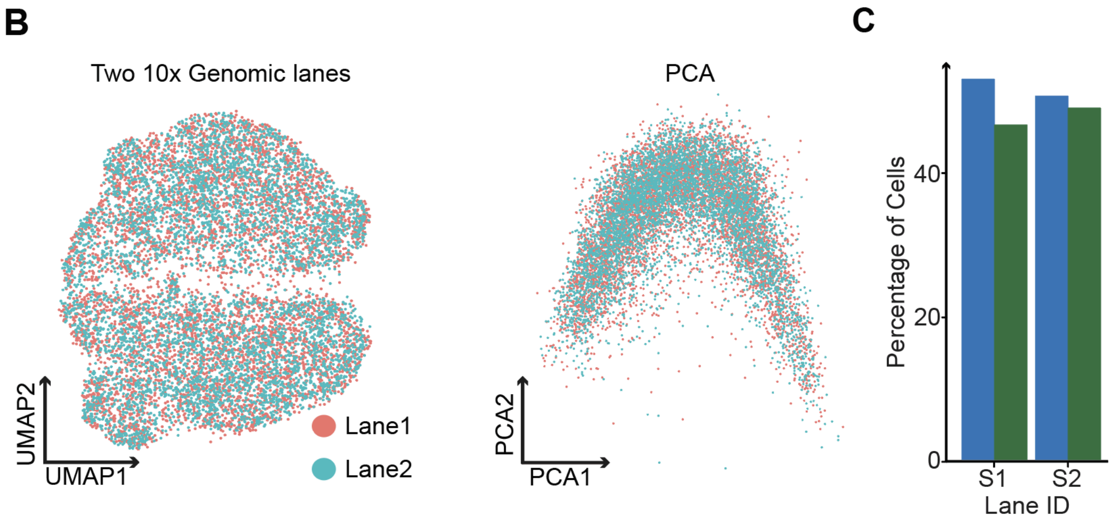

Fig. S5B,C

To further mitigate any potential batch effects and provide rigorous evaluation, we applied "Harmony" before dimensionality reduction and visualization [1]. This method is specially designed for correcting batch effects in single-cell RNA-seq data, ensuring that our observations and patterns derive primarily from the underlying biology rather than procedural discrepancies. Importantly, post the application of Harmony, we observed minimal changes to the PCA scores, indicating the absence of pronounced batch effects due to the 10x lanes. To provide a more quantitative perspective on this, a plot comparing PC1 vs. Harmony_1 showcased Pearson correlation coefficient of 1, and similarly, PC2 vs. Harmony_2 also presented a correlation of 1, Reviewer Figure 2. These tight correlations further solidify our assertion that no significant batch effects were present in our data, and any variations observed can confidently be attributed to biological differences rather than technical anomalies.

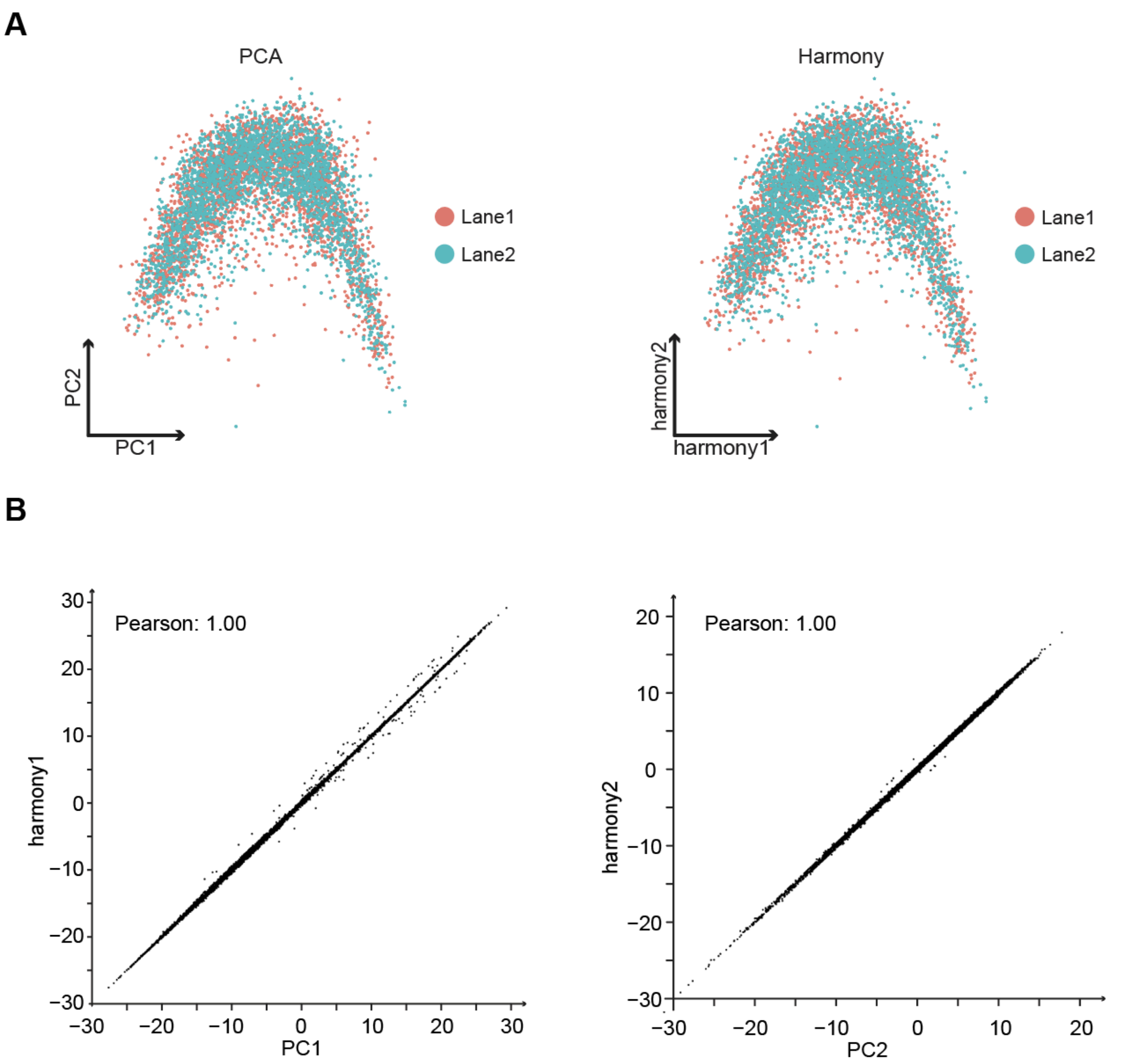

Reviewer Figure 2: Batch correcting measures determine that no significant batch effects are present. (A) PCA and Harmony plots depicting cells processed through two distinct 10x Genomics lanes. (B) Scatter plot of Harmony scores against PCA values.

The reviewer also asks about the fact that we do not see clear clusters designating our proliferative and non-proliferative states. However, this is because the UMAP projection is largely capturing differences in cell cycle. Thus, we regressed out the cell cycle effect (described in the methods: "scRNA-seq cell cycle regression"), and with multiple different dimensionality reduction techniques (UMAP, PCA, t-SNE, Glmpca), we consistently observe distinct clusters representing our highly proliferative and less proliferative clonal states. These plots have been added to Fig. S4C,D and are shown below.

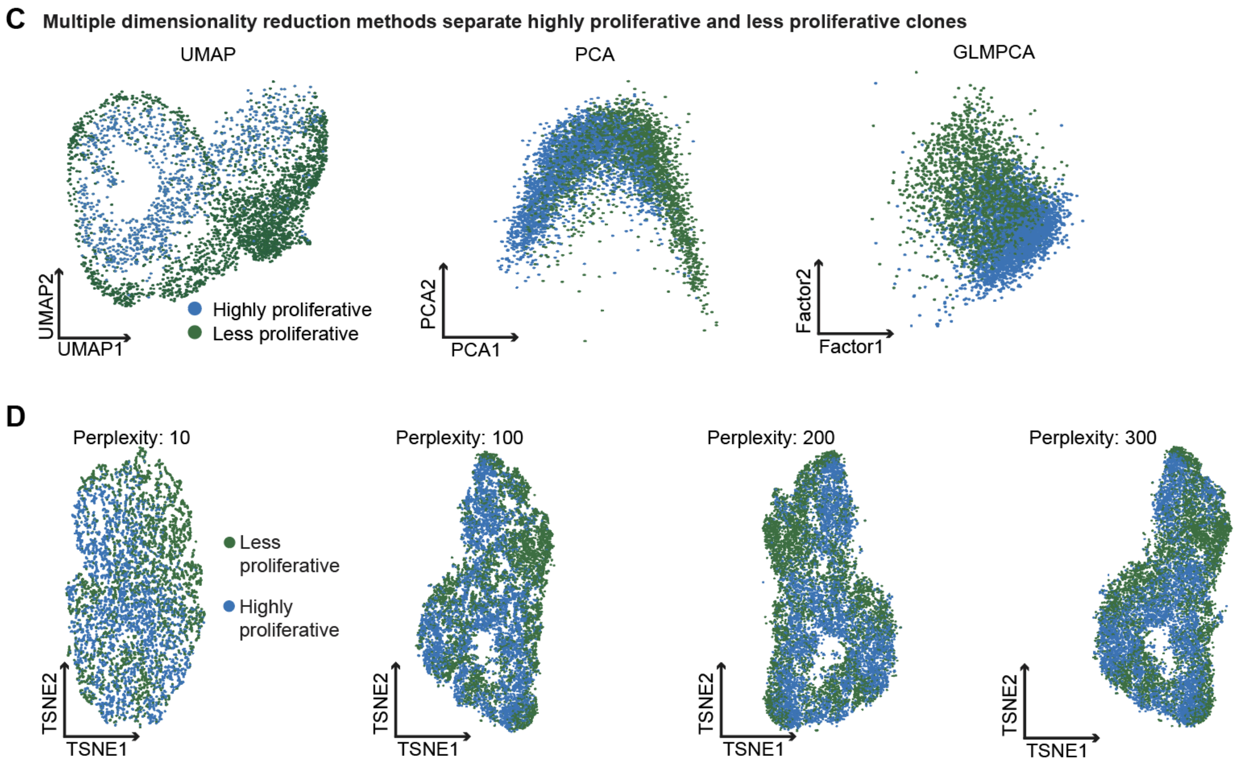

Fig. S4C,D

We acknowledge that it is standard to repeat experiments for reproducibility and have done this for all other experiments in this manuscript and added further details regarding both biological and technical replicates. For the scRNA-seq, we performed two lanes of libraries, but did not perform independent biological replicates given the cost and time considerations of these experiments. Instead, we have validated the key findings from these experiments, including the PI3K activity, Fig. S6E,F, (now Fig. S6A-C), staining for KRT14/Ki67, Fig. 4 A,B and Fig. S5H (now Fig. 4 B,C and Fig. S5E), and CRISPR Cas12 DKK1 KO, Fig. S6A-C (now Fig. S6E-G).

o “We then labeled cells based on their respective clone sizes. Consistent with our previous observations, cells in the top 10% of clone sizes were labeled as expanding, while those in the remaining smaller clones were labeled as committed.” The statement sounds cyclic.
o Fig S4C: need to explain how the expanding colonies are assigned in a quantitative way. See comment above

The cyclic statement you pointed out has been rephrased for clarity, lines 257-261.

We have also reconsidered our analytical approach in response to your insightful feedback and introduced a new method for identifying the clones. We delve into the

specifics in the subsequent comment, highlighting our adoption of cell cycle regression to categorize clusters based on the locations of the largest colonies (lines 271-280).

• Figure 4: A rare subset of cells with an expanding transcriptional signature was found in the human esophageal epithelium
o Fig 4 D and H: the author should clearly state how the expanding signature is selected.

We have updated the manuscript to more clearly explain how we selected the expanding signature and included this in the methods ("*scRNA-seq cell cycle regression* " and "*scRNA-seq analysis and filtering*").

First, we regressed out the cell cycle using specific gene sets for the S-phase and G2/M-phase, as identified in previous studies [2]. Using Seurat's CellCycleScoring function, we scored each cell for these phases. Seurat's ScaleData function was then used to adjust for cell cycle-related variations in gene expression. This allowed us to ensure that our gene expression analysis was reflective of biological changes, not just cell cycle effects. A UMAP projection of the data after cell cycle regression is shown in Fig. 4B,C. Next, we used the number of cells with each barcode to measure the size of each clone in the scRNA-seq data. We then overlaid the number of cells per lineage barcode onto the UMAP (Fig. 4D). This allowed us to clearly visualize and label clusters that belonged to either the Highly proliferative or Less proliferative clones (Fig 4E, below).

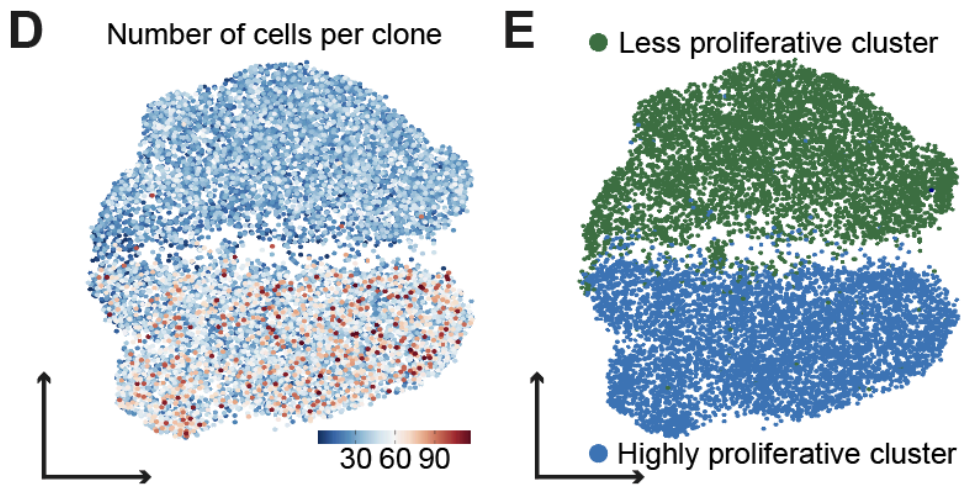

Fig. 4D,E

Subsequently, we conducted a differential gene expression analysis between the highly proliferative and less proliferative cluster. We then determined the highly proliferative and less proliferative gene signatures by using the following stringent criteria: first, we included only those genes with an adjusted p-value of less than 0.05. Second, we set a fold change cutoff, selecting only genes that are a $\log_2$-fold change greater than 0.5

(highly proliferative) or lower than -0.5 (less proliferative). By applying these criteria, we identified 9 genes for the highly proliferative signature and 14 genes for the less proliferative signature. This gave us two unique gene expression profiles, which we used to annotate the highly proliferative and less proliferative clones.

o There are more expanding cells in cancer compared to pre-cancer. This is hardly a noel observation and I do not know what the authors are trying to get at with this human figure. It does not really speak to the inheritability of these states.

Upon revision, we have since reconsidered the framing of this result in our manuscript and updated the final figure to reflect what we believe are the most relevant findings from comparing our results to the in vivo setting. Specifically, we find that less proliferative clones showed higher KRT14 expression, particularly in the morphologically flat and larger cells. This observation, when considered alongside the EPC2-hTERT scRNA-seq and existing literature, suggests that these clones are more likely in a state of quiescence [3–6].

In our revised manuscript, we have recalibrated our claims to align more closely with the central findings of our study. Specifically, we now focus on the presence and implications of the highly proliferative signature in healthy tissue. We believe that this refocused approach offers a more valuable contribution to the field, aligning with our research objectives.

Minor comment

• Please make sure all figure labels are consistent between the text and actual figures. Too many figures mentioned in text do not match the actual figure panels.

Thank you for noticing these discrepancies. We have combed through the manuscript to ensure that all figure labels and references are consistent.

• Reasoning and consistency in units should be specified. For example, Fig2D used 'number of cell divisions' in the plot but used number of cells per clone in text. There is no clarification how the two units are measured/converted.

We have standardized the units used throughout the manuscript and provided conversion methods where necessary. In the example in Fig. 2D (now Fig. 3D) for instance, we added the conversions $\log_2$(number of cells per clone) in the figure caption and explained the reason for this conversion in the methods with the following line (403-405):

“”Clones varied significantly in size, therefore we used a $\log_2$ transformation (referenced as number of cell divisions) to allow for a more effective comparison and visualization of the number of cells per clone that contained vastly different sizes.””

• Color used for each experiment condition should be consistent (see fig S4 B and C)

We apologize for this error. Colors representing experimental conditions have been made consistent across all figures.

Reviewer #2:
Comments to the authors:

The manuscript entitled “Non-genetic differences underlie variability in proliferation among oesophageal epithelial clones” by RA Reyes Hueros et al attempts to provide insight into the molecular mechanism underlying variable proliferation rates for individual clones found in an in-vitro model of oesophageal epithelial cells (EPC2-hTERT) and then aims to find biological relevance by directly comparing the in-vitro obtained data with healthy oesophageal tissue and oesophageal squamous cell carcinoma (ESCC) patient samples.

I would like to start by mentioning that, to the eyes of this reviewer, the observations, experiments and molecular biology concepts described in the manuscript are not novel but rather a re-telling of old school “cell line” cloning observations with a spin of using state-of-the-art tools. It is in that view that, the apparent “novelty” here described has been observed in numerous cases and quite probably is a general phenomenon found in cellular cloning experimentation. The variable rate of cloning efficacy for any given cell line has been well documented and it has been ascribed to numerous cellular and molecular features (cellular crowding, condition media, cell-to-cell communication, cell cycle phase, etc) and by no means is a particular feature of the cell model tested here.

Thank you for the comprehensive and detailed feedback on our manuscript. Below are our point-by-point responses to your comments:

Major comments:

1- There is no description on how the EPC2-hTERT original cell line was generated or how the hTERT was introduced or controlled to increase its expression. Moreover, and extremely important for this kind of studies… was the original cell line already cloned or is it a heterogeneous batch?

We have added details on the generation of the EPC2-hTERT cell line and its heterogeneity (lines 400-403). The integration of the human telomerase reverse transcriptase (hTERT) gene into EPC2 cells is pivotal in preserving their ability to divide, effectively counteracting the telomere shortening associated with repeated cell divisions [7]. Indeed, while it is a heterogeneous batch, our experiments consistently show the same distribution of clone sizes even when starting with a single clone, to better illustrate this we have updated Fig S1.

2- How genetically stable is the EPC2-hTERT cell line? If it is not stable… for how many passages can the non-genetic compartment be interrogated?

The EPC-hTERT cell line was generously provided by Dr. Amanda Muir's lab at the Children's Hospital of Philadelphia. For all experiments in the manuscript, we have used cultures between passages 40 and 48, and thaw a new vial when the cell line reaches this upper limit. We have added this information to the methods section of the paper (lines 412-416). We have not tested beyond this upper limit in passage number as we wanted to control this parameter in our experiments.

More importantly to the conclusions of the paper, we do not see this variability in proliferation change with passage number through our experiments. We consistently find that around 10% of the clones will grow rapidly (either to a much larger size or to fill an entire well of a 96-well plate). Biological replicates derived from different passages of the EPC-hTERT cell line further support the reproducibility of the phenomenon and reassure that it does not fluctuate dramatically with passage.

Related to this point, reviewer 1 also asked about the possibility of genetic drivers of the difference in proliferation. In addition to the reversibility experiments shown in the manuscript in Fig S1C,D, which demonstrates that the proliferative state is reversible, we also performed copy number analysis on our scRNA-seq data (Reviewer Figure 1). From this analysis, we show that the highly proliferative clones do not harbor copy number differences compared to the less proliferative clones (for more details and data on how this was performed see Reviewer Figure 1).

The EPC-hTERT cell line was authenticated by STR profiling. We obtained the expected STR profile for these cells from the lab of Amanda Muir (performed in 2016). We independently performed STR profiling on the culture passage 46 in our lab in 2022. Comparison of the overlapping loci showed a 100% match between the profiles. Data is available on the google drive link for the manuscript.

3- Schemes depicting potential experimental outcome must not be part of figures as they already suggest the results of the experiment prior to the actual evaluation of the data. Moreover, they suggest the possibility that the author contemplated all possible scenarios whereas the authors may be mistaken in their observation and analysis. Please revise all figures depicting schemes for data outcome.

We agree with the point regarding the use of schemes in figures and have revised them to ensure clarity without biasing the interpretation of results. We have removed the boxes that point the reader to specific hypotheses. We have used schematics to outline the experimental designs.

4- As in 3-, though the authors in the text contemplate a "minimalistic" model of proliferation they are leaving out multiple possibilities such as cell death (any kind), senescence, cell cycle

progression speed among others. Even if the model turns out to be the right one, possibilities must be explored and discussed.

The reviewer is correct that our model has limited parameters. We have updated the text to discuss other possibilities and assumptions made by our model (lines 164-168). From our experimental observations using time-lapse imaging, we do have evidence that cell death is minimal supporting this assumption. Senescence is considered in our model as we do have a non-proliferative state; however, return to proliferation is not. We could certainly have decided to add additional parameters to the model, which would, of course, improve fit, but given their unlikely contributions, we aimed to balance between model simplicity and biological reality.

5- In the first bit of the manuscript (Figure 1), cells are fixed and imaged. Therefore, the clones analysed on every time frame are different. I suggest the authors to re-run this experiment but this time following the same clones over time by live imaging. In this way, many additional layers of information may be also extracted from the data and used to complement the model.

As suggested by the reviewer, we have added new experiments to complement our multi-time point fixation experiments with live imaging to track the same clones over time. We performed live-cell imaging using both the mCherry-labeled EPC2-hTERT cell line and wild-type cells. Live-cell imaging corroborated the growth trends observed in the multi-time point fixation experiments. We specifically observe a subset of clones that grow much larger than the others, consistent with our highly proliferative clones captured by other techniques. These live imaging results have been integrated into the revised manuscript, supporting the validity of our original model (Fig. S2E-F). However, we did notice that both mCherry-labeled and wild-type cells generated smaller clones when compared to our non-imaged controls. We also directly compared the live-cell imaging to our cell fixation data and can clearly see a decline of exponential growing clones, with clones ranging from 10-29% less cells, Reviewer Figure 3A,B. Because the clones were significantly smaller, we were not able to integrate this data into our model. We hypothesize that the added stress from growing in the live-cell incucyte and imaged periodically slowed the growth of all clones. This caveat has been added to the methods.

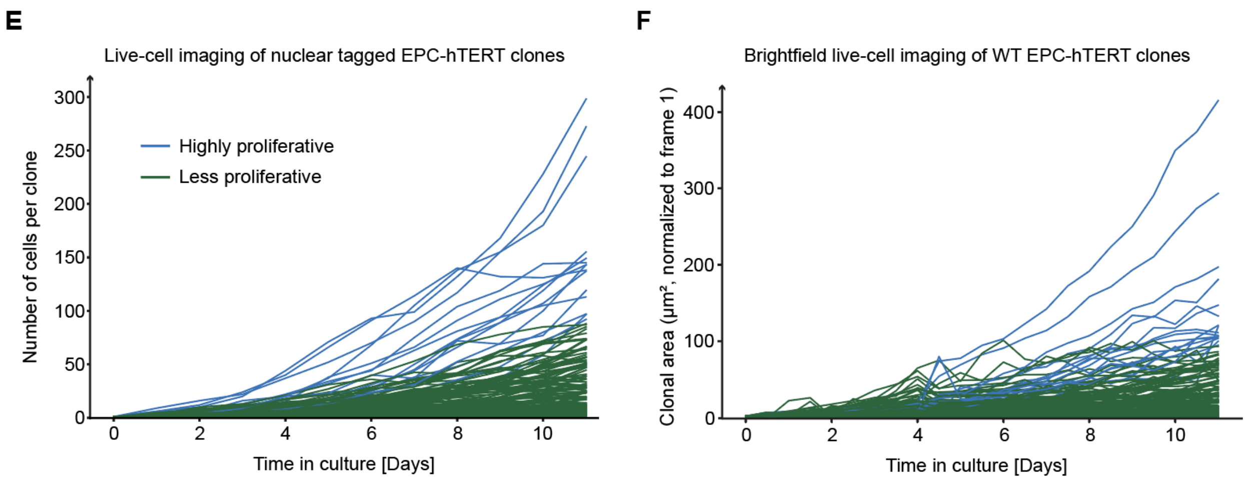

Fig. S2E,F

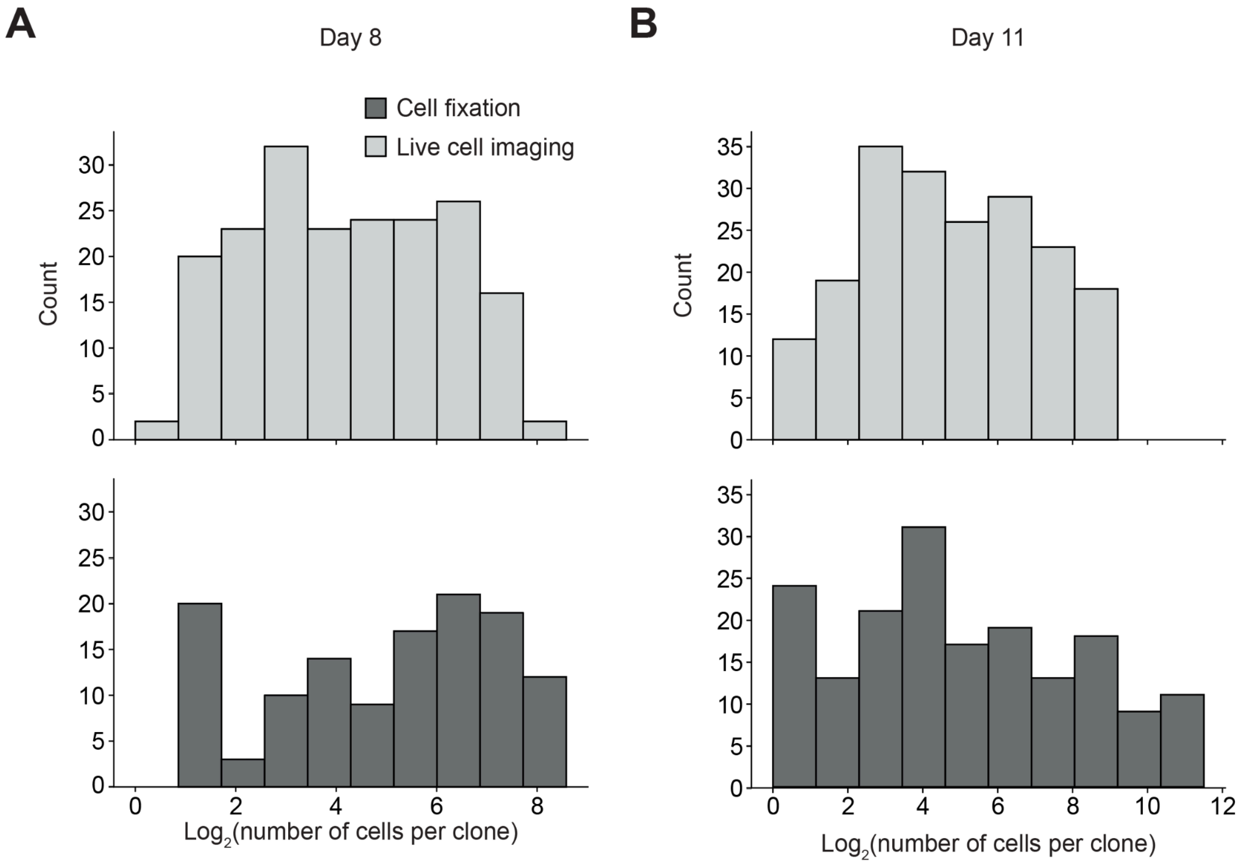

Reviewer Figure 3: (A-B) Histogram of the log2(number of cells per clone) separated by the cell fixation and live cell imaging datasets (day 8 on the left and day 11 on the right).

6- Also, how many times the plating and counting has been made for each data point? How many technical and how many biological replicates? Which is the variance between plates? Is the count based on cell counting or colony size? Is there any kind of filter used to remove small colonies? Are small colonies taken into account at all?

We have updated the manuscript to add the information requested by the reviewer providing a detailed breakdown of the plating schemes, number of technical and biological replicates and counting techniques. Additionally we added a detailed methods section for “multi-day clonal fixation experiment”.

Briefly, cells were seeded in 6-well plates at a density of 50 cells per well and cultured for durations of 2, 5, 8, or 11 days (Fig. 1C and Fig. S2A), and also for 2, 4, 6, 8, 10, or 12 days across 2 biological replicates with 6 technical replicates for each time point. The culture medium was refreshed every four days. At the end of each designated time period, we proceeded with cell fixation followed by DAPI staining. The wells were then imaged using a Nikon Eclipse Ti2 fluorescence microscope equipped with a 10x objective. The number of cells per clone are determined by the number of nuclei detected within the clone. Clones with one cell were filtered out of the analysis.

7- Which is the number of cells or range that the authors used to define highly proliferative from non-proliferative? Is this arbitrary or was model based on the data? If arbitrary, make it explicit in the main manuscript (the authors suggest 10% in the manuscript but further explanation is needed). Was this consistent among biological replicates analysed in the same experiment/across different experiments?

We define the highly proliferative clones as the top 10% based upon experiments (Fig. S1A,B) in which we plated single cells into 96-well plates and observed that 10% of these grew to the point at which they filled a well. We used this percentage going forward as we saw that it was consistent with the largest clones (which were still quite distinct from the others) in the 6-well plate format as well. We have revised the manuscript to make this methodology clearer (lines 97-102).

8- Do smaller (in number of cells) clones stop proliferating or their rate of proliferation is reduced? This can be also analysed by live imaging.

The reviewer raises an interesting point here about the need to clarify the behavior of smaller clones in our study. Overall, we do not claim that these small clones completely halt their proliferation. Even in the single-cell RNA-seq data in Fig 3D, we observe a subset of small lineages that fall within the transcriptional states that are consistent with cycling cells.

Furthermore, as this reviewer suggested, we used live cell imaging, as previously described in comment 5 above, to monitor the growth of these clones. While overall the

live-cell imaging does induce additional stress, and thus, somewhat alter the growth patterns of the clones, we still observe slow growth (compared to the highly proliferative) among smaller clones on the plate. We also detected clones that completely stopped proliferating in each replicate, 9.8% and 20.3% respectively (Reviewer Figure 4).

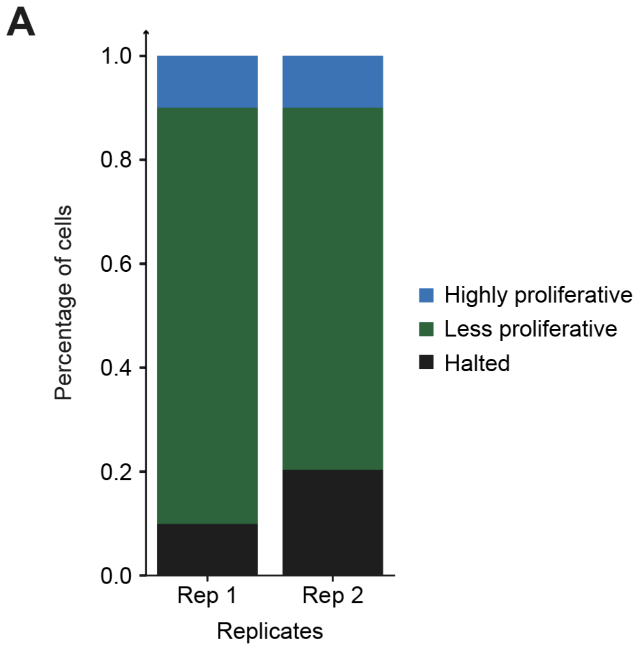

Reviewer Figure 4: (A) Barplot showing the percentage of cells in highly proliferative, less proliferative or halted state, split by replicates. Here, halted state refers to clones that stop growing completely between days 8 and 11 of time lapse imaging.

9- Certain definitions presented here needs to be expanded and explained in the context of the manuscript… For instance, "… We included the non-proliferative state because some clones contain cells that stop proliferating and show morphological features consistent with differentiation…". Differentiation into? Also, a change in morphology does not necessarily mean "differentiation". What do the authors imply here? I suggest the authors to clarify the concept.

We found that most clones largely stopped proliferating by 8 days and displayed distinct morphological changes, including lower expression of Ki-67 (Fig 1D). We have clarified these observations in the text and removed the use of the term "differentiation".

However, to add to our interpretation of these morphological observations, we have added new experiments in which we stained these clones for Ki-67 and KRT14. We selected Ki-67 to label cells from our highly proliferative clones as this is a canonical marker of proliferation. Based upon our scRNA-seq experiment, we also identified

KRT14 as highly expressed in the smaller clones in our data, which is a marker of quiescent basal cells in the human esophagus.

Consistent with our predictions, we found that the less proliferative clones exhibited expression of KRT14, specifically in the morphologically flat and larger cells within these clones (Fig. 5B). In the context of the literature [5,6,8,9], the expression of KRT14 would suggest that these less proliferative clones are in a state of quiescence rather than actively undergoing differentiation into more specialized cell types typically seen in the stratified layers of the esophageal epithelium.

We have updated the manuscript to reflect these findings and provide a clearer understanding of the nature of the less proliferative clones in our esophageal epithelium model (Fig. 4) (274-291).

10- Figure 1c is not clear and the legend does not help either.

We thank the reviewer for this insight. We have made Figure 1C more clear by modifying the y-axis to solely represent the frequency of cell divisions. We then added a legend to represent the total time in culture (2, 5, 8, and 11) and then have explained this further in the legend with the following text:

“”Histogram of the number of cell divisions, $\log_2$(number of cells per clone), separated by days in culture, as shown by the colors in the legend. The gray vertical lines show the cell number if they doubled every 24 hours (n=2 biological replicates each with six technical replicates, one biological replicate shown with all technical replicates).”

11- I think that definitions here are needed. For instance, “… Thus, we separated the clone size data (Fig. 1C) into the top 10%, which we named "expanding". For the remaining clones, we assumed that they followed a pattern consistent with the halting of proliferation, so we named them "committed"…” . Committed to what? The wording here needs to be revised as the word “committed”, and depending on the field of research, alludes to multiple biological scenarios (determination, differentiation, lineage, etc).

We appreciate the reviewer drawing our attention to the possible issues with the terminology of “committed”. We agree that the word “committed” has been attributed to multiple biological scenarios and for those reasons has been changed to “less proliferative” as these clones have limited capacity for proliferation. We have also revised the term “expanding” changing it to “highly proliferative” as this term more accurately describes unique characteristics of these clones. Thus, the two clone types are highly proliferative and less proliferative. We have updated the manuscript throughout using this new terminology as it is more specific to our experimental observations.

12- In the experiment of the 96-well plate… How many clones were plated? How many clones survived? How many “stop” proliferating after several cell divisions? How many did not grow at

all? How many were retrieved? How many wells were empty after the sorting? How many times the experiment was carried out? What happens if you re-plate the clones that displayed halted proliferation? All this data cannot be neglected as all these potential phenotypes contribute to the overall outcome of the population.

We thank the reviewer for highlighting some information that would benefit the reader of this manuscript. We have added new plots in Fig. S1, that explicitly show the number of cells plated and the number of clones surviving. Given the experimental design here, it was tricky to make accurate measurements of single-cells or dying clones. To better do this, we performed live-cell imaging experiments to better quantify some of these other questions; however, our live-cell system led to altered growth dynamics likely due to the stress of repetitive imaging. In our live cell data, we did quantify the clones that have stopped proliferating and found only a subset of less proliferative clones stop proliferating (also addressed in comment 8).

The 96-well plate experiment in Fig. S1 was performed with 3 biological replicates derived from different passages of the line on separate dates, each of which consisted of 3 technical replicates consisting of different 96-well plates.

We did try to re-plate the clones that stopped proliferating (using three technical replicates Supplementary Table 1), but never generated a highly proliferative clone or even one with minimal growth. This would suggest that the less proliferative state can not switch back into the highly proliferative state. However, we are cautious with this interpretation as these experiments contain far fewer cells (the initial clones are very small). Given the interest of this reviewer and reviewer 1, we have added information about this experiment and its results to the manuscript (lines 133-134).

We also replated seven highly proliferative clones, each with varying numbers of replicates (three biological replicates with 3, 2, and 2 technical replicates respectively), The subclones originating from the initially highly proliferative clones displayed growth patterns akin to their original populations.

13- In the following paragraph, "… If genetic differences in these clones were responsible for the growth differences, we would expect that all of the subclones derived from the highly proliferative parent clone would grow similarly to the parent. Instead, we found that the subclones followed a similar growth distribution to the initial population (Fig. S1A). Thus, we can conclude that the highly proliferative clones are not a genetically distinct subset of the EPC2-hTERT models…" the authors suggest that the observed phenotype is non-genetic as it re-shuffles to the original proliferation distribution observed in the population. However, and as stated above regarding the novelty of the study, this feature is quite probably a general phenomenon found in cellular cloning that can stem from a multitude of biological inputs. I suggest the authors to tone this down.

We agree with this reviewer that this phenomenon would likely be seen with other cell lines cloned in this fashion and have cited relevant literature on the topic. We have also

further emphasized precisely what we can say from these experiments, which is that the proliferative phenotype is reversible in nature. We also agree with this reviewer that other biological inputs can govern growth and discuss such factors and explore the role of cell density in Fig. 3C,D. We also explore the molecular differences between these highly and less proliferative clones using scRNA-seq and barcoding.

14- In Figure 2… it is my opinion that the data depicted here directly contrasts the data shown in S1A. The authors suggest a "… When we compared the number of reads in split A and split B, we found a significant correlation in the number of reads for each barcode across the different plates (Fig. 2B)…". However, if the highly proliferating clones, once plated at single cell confluence re-constitutes the initial grow heterogeneity it should be expected a 90% "committed" (not the right wording here) and 10% expanding… how does the authors explain the high correlation observed? The barcodes, used as proxy for cell proliferation here, should also display the same behaviour. I suggest the authors to look deeper into the "barcodes" dataset as to this reviewers' view, both experiments should show, if the authors are right, a degree of concordance.

The distinction here is in the number of divisions. In our 96-well plate experiments (Fig. S1B), we allowed the cells to grow for 14 days, resulting in > 10 doublings. Over this time, the memory of the growth potential is lost, and the cells from the same clone have diverged in their growth potential. In Fig. 2B (now Fig. 3B), we limited the growth of the initial clones to 4 doublings and then we split across conditions. On this shorter timescale, we observe that the growth potential is partially heritable in the cells derived from the same parent cell. We have discussed these experimental differences and their interpretation in the text to clarify this for the reader (lines 173-178).

15- Also, in Legend to figures, especially in Supplementary Figures, there is a lot of description and interpretation of the results. This must be removed from legends to figures. Legends should only describe the data depicted and the way the data is represented.

We revised the figure legends as outlined by the reviewer to describe the figure and data.

16- Also, in relation to data and Figure 2, it would be useful to describe how many barcodes where detected, how much data was removed, how many cells are estimated to be properly labelled (1 copy per genome), etc. The inclusion of those metrics will help the potential reader to better evaluate the dataset.

Detailed metrics on barcodes have been added to the methods. Reviewer Figure 5 (seen below) shows the distribution of the number of detected barcodes per cell. A large portion of the population has a single barcode (~50%), with a steep decline. After filtering for cells without barcodes, the dataset consists of 15,552 cells. Of note is the large percentage of cells with no barcodes, which is at least partially due to limited capture efficiency with scRNA-seq for the barcode transcripts. This aspect of the data has been

made explicit in the methods. The clonal analysis of the scRNA-seq data only includes cells that have the lineage barcodes, therefore, cells containing no barcodes (7893 cells) were discarded, as well as cells with four or more barcodes (228 cells).

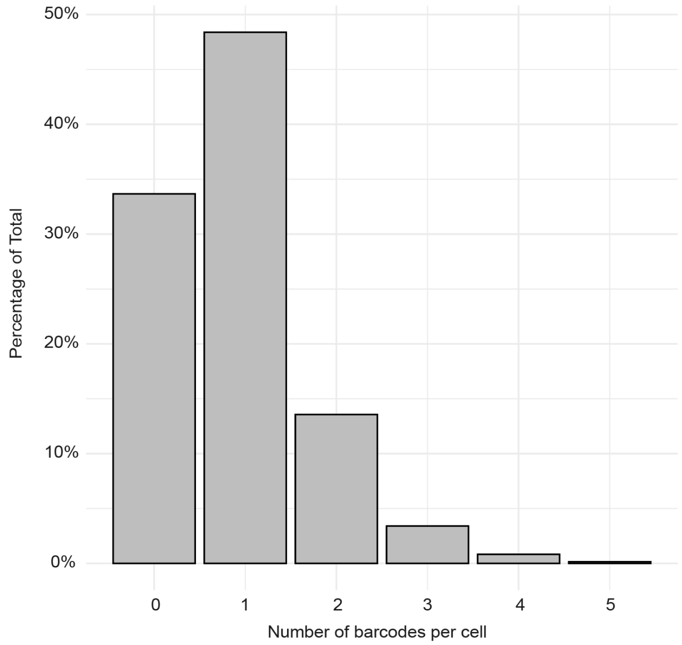

Reviewer Figure 5: Percent of total barcodes detected per cell

17- In Figure 2b. It reads “Barcodes show memory of proliferative capacity”. Barcodes are not showing anything… The cells expressing/labelled with a given barcode suggests “something”.

We agree with the reviewer regarding the incorrect wording for this label, and have removed it. In Fig. 2B (now Fig. 3B), we changed the analysis to more explicitly test for whether the different clones labeled by the barcodes are highly proliferative versus less proliferative. Now, the label states “Comparison of experimental data to simulations” as this panel shows the experimental data from two replicates and simulated outcomes if the proliferative state does not have memory.

18- In Figure 2c the authors attempt to explain the effect of cell number in the proliferation capability for clonal growth. Once again, this is not novel and it has been shown endless times. The more cells you have on a plate whilst not in confluence conditions, the better cells grow and proliferate.

We understand the concern about the novelty of the findings in Fig. 2C (now Fig.3C). We provided appropriate citations and adjusted the text to reflect this (lines 220-222) We have also reframed the question in Fig. 2C (now Fig.3C) to emphasize why we did this experiment and how it teaches us more about the phenomenon. Given that increasing the density of cells is generally known to improve growth (up to a point at which other

resources are limiting), we wondered whether such conditions would allow all clones to grow to similar sizes or whether clone size would still show differences. For instance, increasing density could allow every cell to have an increase in its growth rate, but start from some baseline, which we observe in the clonal plating experiments. In this case, we would expect to see a broad distribution of clone sizes (although an overall increase in growth). Alternatively, increasing the density of cells on the plate could allow every cell to grow at a similar rate. If this were the case, we would expect to see a more uniform distribution of clone sizes. In Fig. 2C (now Fig.3C), we find the second hypothesis, that most clones grow to similar sizes over 8 days and the clonal differences are effectively erased.

19- Regarding the single cell experiments depicted in Figure 3. It is not clear to this reviewer how the experiment was performed. The figure suggest that the clones are collected based on the number of cells (e.g CellCelector?) and then pooled? Or is it just clonal growth by dilution and then the plate is collected and processed for single cell analysis? The distinction is fundamental as, based on the results shown here (e.g. Fig. 3E), many more cells from the expanding clones should be sequenced when compared to the "committed lineage" found in the plate which would be minimal in the contribution to the overall pool. By analysing the UMAP depicted in Fig. 3D this does not seem to be the case and, moreover, it looks like there are more "committed" cells than proliferation ones. This reviewer may be missing something here, but the data depicted is telling a contrasting story. Could the authors please develop?

We thank the reviewer for pointing out some information that could be added to this figure and legend. Every cell is labeled with a unique barcode that is in a lentiviral backbone such that it is incorporated into the DNA of the cells. When the clones proliferate, all progeny from that same parent is labeled with the same barcode. We then pool all of these cells together (containing many different clones) and perform scRNA-seq together. We have updated the schematic in Fig. 3A (now Fig.4A) to clearly point out these steps.

This experiment was performed on day 8, so that the differences between highly proliferative and less proliferative clones are less exaggerated in their sizes. In the multi-day fixation experiment, Reviewer Figure 6A, you can see that the top 10% of largest clones take over about 50% of all cells by day 8. As the reviewer has pointed out, the total number of cells between highly proliferative and less proliferative cells in the scRNA-seq data is skewed in favor of the less proliferative cells, Reviewer Figure 6B. Specifically, we see about 25% highly proliferative by scRNA-seq versus 50% in our fixation experiments (Reviewer Figure 6B). We think these differences are minimal and probably reflect experimental differences. Importantly, the proliferative cells are growing at an exponential rate, so a small deviation could cause large growth differences as seen here. It is also possible that stress induced by the experimental design, including barcode transduction and FACS sorting, could cause these cells to proliferate slower.

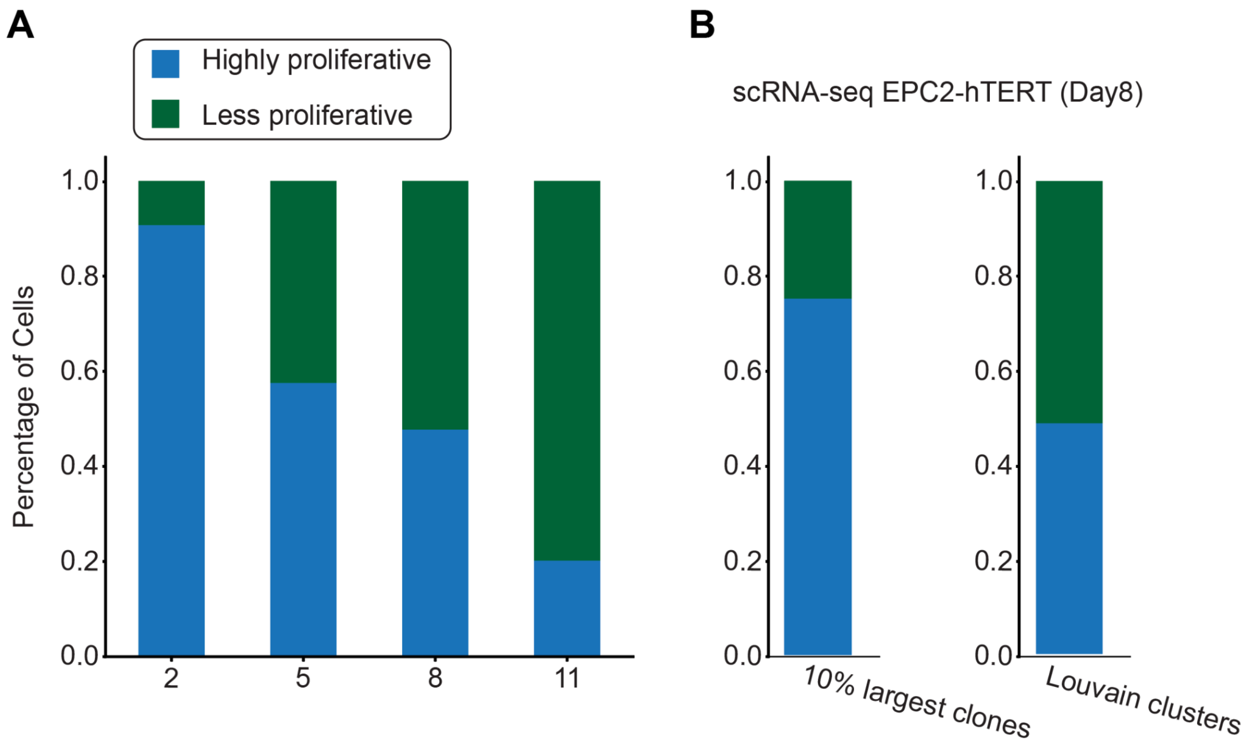

Reviewer Figure 6: Distribution of highly proliferative and less proliferative clones by imaging and RNA-sequencing. (A-B) Stacked bar plots showing the percentage of highly proliferative and less proliferative cells separated by time in culture. On the left, data is from the multi-day fixation experiments in Fig. 1. On the right, data is from the EPC2-hTERT scRNA-seq dataset in Fig. 4.

In a related analysis, we used our barcodes to identify the highly proliferative and less proliferative signatures (as described in the methods "*scRNA-seq cell cycle regression* " and "*scRNA-seq analysis and filtering*") then we applied these signatures to the louvain clusters of cells in our dataset. In these clusters, we see about 50% of cells in the highly proliferative state and 50% in the less proliferative. This corresponds to cell state, not barcodes (Right bar plot, Reviewer Fig. 6B).

20- Also, and given the data displayed in Figure S1A, phenotype interconversion is happening. It would be informative if the authors would explore the data for cells that belong to a given "lineage" that are actually changing their phenotypic output. Please provide numbers and discuss possibilities.

The reviewer raises an interesting point regarding lineage-specific phenotype interconversion in our scRNA-seq data. We have delved deeper into our scRNA-seq data to analyze cells within specific lineages and their potential phenotypic interconversion. Through the barcodes, we determined that most clones are in a single state, with 547 clones in a highly proliferative state and 790 clones in a less proliferative

state, (Reviewer Figure 7A). We also determined 566 clones to have a mixture of these two states (Reviewer Figure 7B).

We then measure the extent of gene expression levels differences between the three states. Specifically, we compared cells from lineages that are in a mixed state of highly proliferative and less proliferative cluster against those that remained within a single cluster, Reviewer Figure 7C,B. No discerning genes were detected through this analysis. All genes that passed the threshold criteria in either comparison were also found in our direct comparison of the highly and less proliferative states in Fig. 4. This suggests that the mixed lineages do not have their own unique signature, but rather they are just a mix of the other two. This approach allowed us to discern and quantify the degree of phenotype interconversion, providing a clearer understanding of the dynamics within these cell populations.

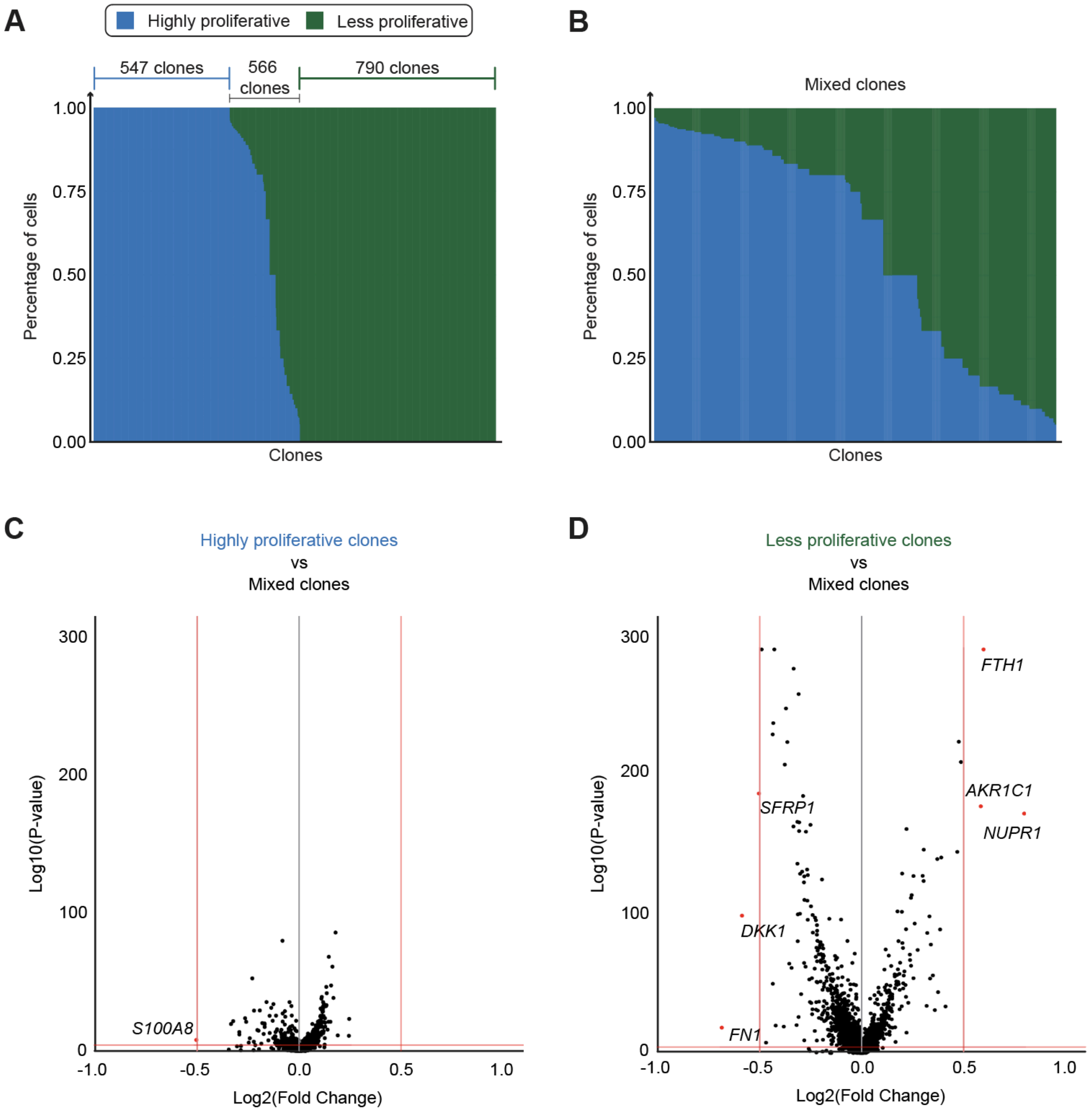

Reviewer Figure 7: Distribution of highly proliferative and less proliferative clones as determined by scRNA-seq barcodes. (A) Bar plot showing the percentage of each lineage organized based on their percentage of highly proliferative clones. Each bar represents an individual clone. (B) Bar plot highlighting the clones that transition from panel A. (C) Volcano plot of differential expression analysis between highly proliferative (positive fold change) and transitioning clones (negative fold change). (D) Volcano plot of differential expression analysis between Less proliferative (positive fold change) and transitioning clones (negative fold change).

21- The authors are measuring “steady-state RNA levels” and not “active transcription”… Therefore, the authors should not argue that they are observing “transcriptional differences” but rather “gene expression levels differences”. The difference is not un-important.

We acknowledge the distinction between "steady-state RNA levels" and "active transcription". To address this, we modified our terminology from “transcriptional differences” to "differences in gene expression" as advised and have adapted the text.

22- Information about cell cycle embedding for the single cell analysis is not available within the manuscript. How was this performed? How many gene expression markers were used? What is the underlying theory behind the colour gradient code? I have serious doubts about inferring cell cycle transitions using gene expression profiles… cell cycle phases yes… cell cycle transitions… not sure at all. Please develop the framework.

Thank you for your comment regarding our cell cycle embedding analysis. We utilized the tricycle package for a more accurate cell cycle position prediction in single-cell RNA-seq data [10]. Tricycle integrates biological aspects of the cell cycle with mathematical principles of principal component analysis and transfer learning. By estimating cell-cycle embedding from a fixed reference dataset and projecting new data onto this embedding, tricycle offers robust predictions of cell-cycle positions across various datasets, our data is shown below, Fig. S4C (now Fig.4E). This analysis was performed on the data set prior to cell cycle regression (as we aimed to show cell cycle effects). However, Fig. 4 has been updated with UMAP plots after cell cycle regression as we used this to derive the transcription signatures of the different clone types.

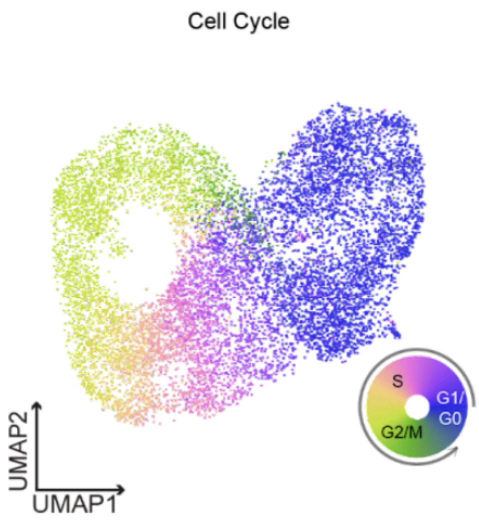

Fig. S4E

The tricycle package reduces the dependence on selecting specific gene markers. However, We identified particular gene sets representative of the S-phase and G2/M-phase, referencing gene expression patterns previously documented in literature

[2]. By employing the CellCycleScoring function in Seurat, we were able to allocate scores to individual cells according to these gene sets, thereby determining their respective cell cycle phases (Reviewer Figure 8). This alternate method correlates fairly well with the tricycle embeddings.

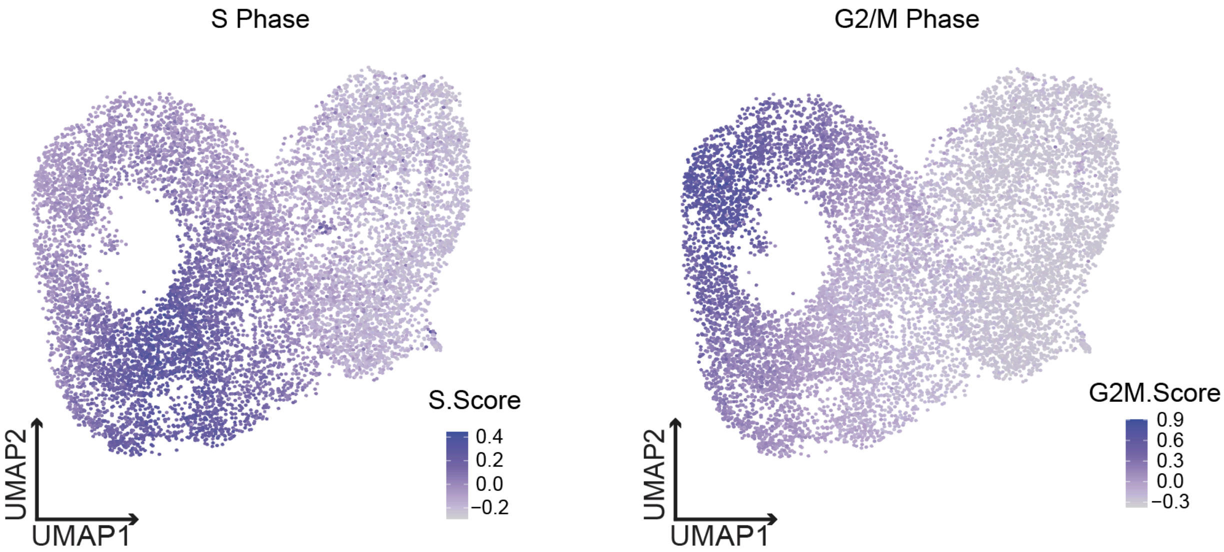

Reviewer Figure 8: Cell cycle scoring of the S-phase and G2/M-phase.

We have incorporated a more detailed description of the rationale in the methods section of our manuscript.

23- Regarding the gene expression signature associated to proliferative clones found in the in-vitro system and then tested with datasets derived from healthy tissue and patient samples… More information must accompany the analysis of these datasets. Is the data batch corrected somehow? is the data “integrated” somehow? If so, which are the anchors and by which method was this achieved.

We have added a comprehensive description in the methods section regarding the processing of patient datasets.

In summary, each dataset was independently normalized to correct for sequencing depth and technical biases, ensuring comparability across different sources. We performed doublet detection to identify and exclude potential doublets to each dataset separately, enhancing data accuracy. Cell types were annotated within each dataset based on known gene expression profiles for accurate comparisons. To address batch effects, we improved the alignment of cell clusters in a shared PCA space, utilizing RunHarmony based on the samples' patient identification. This method is intended to minimize technical discrepancies without obscuring biological variations.

Cells types, labeled across different datasets, consistently clustered together after batch correction. This structured approach allowed us to combine datasets effectively for investigating the gene expression signature associated with highly proliferative clones.

24- Regarding my previous comment… at least 1/3 (perhaps a little bit more) of the ESCC patients do not follow the signature. Do the authors have access to the clinical features from each sample? Perhaps the signature can be further refined. Also, Fig. 4A shows 24x Patients and I count 26 bars in the histogram (healthy? Or tissue surrounding ESCC?). Similarly, Fig. 4E states 55x Patients… I count 64 bars in the histogram. Also, it would be interesting to include examples of patient samples from both extremes and show them in contrast to healthy samples. A few UMAPs would be informative here.

Unfortunately, we do not have access to clinical features associated with these public datasets. Given multiple concerns from the reviewers and our limited claims from these analyses, we have removed the comparison to cancer samples. Rather, in Fig. 6, we have focused on these signatures in the normal esophageal epithelium using the healthy dataset [6,11–13]. We asked whether the highly proliferative clone signature is expressed in the normal esophagus, and if so, which cells express this signature? Indeed, we found the highly proliferative signature is expressed in the normal esophagus and is most enriched in a suprabasal subset of cells that are actively dividing.

Although the analysis in question has been removed, we would still like to address the discrepancy between patient numbers and bars in the histogram. The bars were accounted for by each patient, the issue is that some patients had multiple biopsies (samples) taken and we only added the number of patients instead of the total number of samples. This occurred more frequently in ESCC patients as the reviewer accurately points out.

25- Also in relation to my previous point, I've just roughly calculated the number of cells per patient used in the previous analysis and I've found that for healthy tissue you'd get ~3000 cells per sample (patient) whereas in the ESCC panel you'd only get ~441 cells per sample (patient). Will that be sufficient to support the conclusions here drawn? It would be informative to explore the integration of both datasets Healthy vs ESCC. I must stress that I'm a little sceptical about the validity of this comparison given the divergence in the number of cells in both datasets. Please develop and include metrics for all this.

We agree with the reviewer that there was a challenge with our prior analysis given the differences in these dataset. We have outlined in the methods the challenges and caveats of comparing these public datasets. As mentioned above, given the valid concern of the reviewer we have chosen to focus on the healthy dataset as this allows us to more clearly relate the patient data to our model system.

26- Looking at the data suggesting that markers of highly proliferating cells can be derived from the in vitro system, this reviewer keeps wondering whether the signature is nothing else but a proliferation signature that can be found pretty much in every proliferating cell line? How many

markers are used to define highly proliferating cell from healthy and ESCC patients? I find that, defining a population by the expression of a few transcripts, especially from tissue samples, may be misleading. This information/concepts must be expanded in main manuscript.

We agree with the reviewer regarding this point. We have removed the analyses of the ESCC patient scRNA-seq from the manuscripts and focused our claims around the cell line and normal esophagus.

27- The finding that WNT and PI3K signalling pathways are altered in highly proliferating cells is not surprising as it has been reported numerous times. Therefore, this reviewer finds the experiments of CRISPR KO and PI3K inhibitors not informative enough to make a step forward into describing the molecular mechanism. Also, upregulation/downregulation of a transcript does not necessarily mean enhanced translation of the corresponding protein. Therefore, I suggest the reviewers to tone down all claims directly linking transcript modulation with the presence/absence of a protein/pathway.

Given the established understanding of the WNT and PI3K signaling pathways, we have tempered our claims. We acknowledge the gap between transcript modulation and actual protein presence and have updated our assertions.

28- The discussion must be toned down as to describe what the manuscript is actually showing.

We have revised the discussion section of our manuscript to more accurately reflect the specific findings and conclusions drawn from our data.

Minor comments:

1- The manuscript must be revised for consistency. The wording must reflect the actual data.

The manuscript has been revised for consistency, ensuring the wording appropriately represents our findings.

2- Figure legends must be re-shuffled to not include explanatory text.

We revised our figure legends to focus solely on describing the data, excluding any explanatory text.

3- The methods section must be further developed to include every single experiment and analysis so any given reader can reproduce the results here presented.

The methods section has been expanded to detail every experiment and analysis for replication purposes.

4- This may be a consequence of file conversion but, Figures are fuzzy. Also, please work on the figures to make them auto-explicative.

> We believe the fuzziness may have been caused by the file conversion process. To address this, we have re-rendered all figures at a higher resolution and refined the legends and labels to ensure each figure is clear and self-explanatory.

6- In Supplementary Fig.5 the cells labelled as expanding in the UMAP seems to be bigger (as in bigger dot) than the ones in the markers UMAPs. This representation seems a little misleading to this reviewer. Please make it uniform.

> To address the disparity in dot sizes in Supplementary Fig.5, we made them uniform, ensuring a consistent representation across UMAPs.

Reviewer #3:
This is an interesting manuscript that address the important question of the contribution of non-genetic mechanisms to differences in growth potential. By combining scRNA-seq with barcoding in an in vitro model of esophageal epithelium, they show that non-genetic proliferative capacity in a heritable trait that can be influenced by extrinsic factors such as cell density. Focusing on highly proliferative clones they found a cellular state which is associated with upregulation of the WNT and PI3K pathways. Finally, they show that this proliferative cellular state can also be found to some extant in patient data.

I think this manuscript address a fundamental largely overlooked aspect of non-genetic variation however there are several points that needs to be addressed to make the claims stronger, namely:

> We greatly appreciate the reviewer's time and thoughtful feedback on our manuscript. Please find our responses to each comment below:

1) The author propose an that what they findings can not be explained by "a simple two-parameter model of self-renewal and differentiation" however throughout the manuscript the two main phenotypes are proliferative and arrested. If the authors could show that the smaller colonies are showing distinct markers of differentiation, I think that could significantly contribute to their work.

> We appreciate this recommendation and agree that demonstrating distinct markers of differentiation in smaller clones could strengthen our argument.
>
> To address this, we have expanded our analysis by incorporating immunostaining for Ki-67, a canonical marker for cell proliferation, and KRT14, a marker indicative of quiescent basal cells [3–5,14]. This target was selected from our scRNA-seq analysis as it was enriched in the less proliferative clones (Fig. 4A). Using immunofluorescence, we show that the smaller clones, which we previously categorized as 'less proliferative

clones', stain positive for KRT14, which is a marker of quiescent basal cells rather than stratified, differentiated epithelial cells (Fig. 4B). This observation suggests that these clones are more likely in a state of quiescence, rather than actively undergoing differentiation into specialized cell types typical of stratified esophageal epithelium layers.

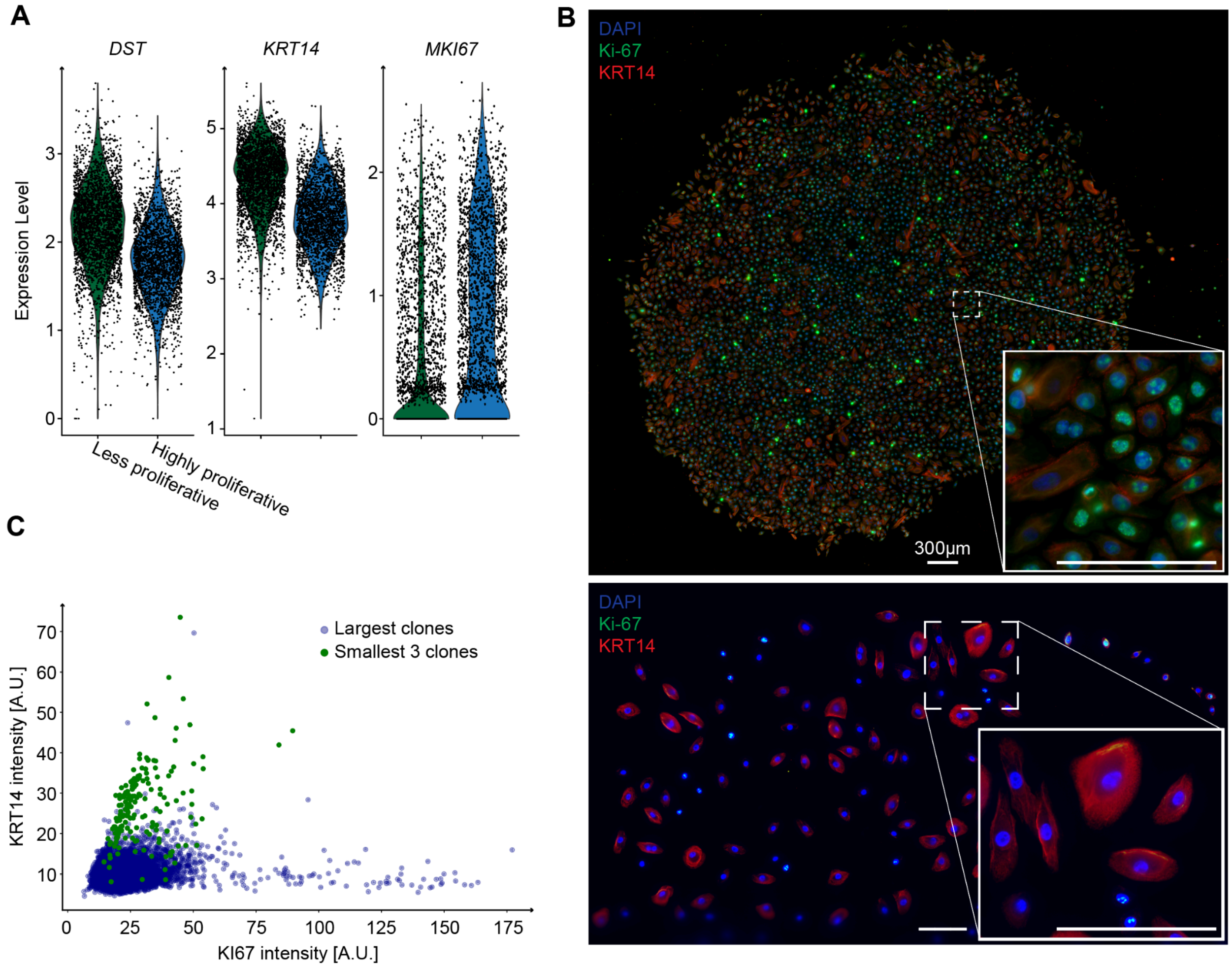

Fig. 4

The relevant text and figures detailing these results can be found in lines 310-329 and Fig. 4 of the revised manuscript.

2) I would be good to calculate what are the transition rates between the two states defined above.

We agree that it would be informative to calculate such a transition rate. For this calculation, however, we needed to collect additional data of the same clones through time. Our experiments in Fig. 1A are from different plates fixed through time, thus complicating using this data for a model. Instead, we performed time lapse imaging such

that we could track the growth of a single clone through time. However, after collecting this data (now in Fig. S2E,F), we realized that the overall clone size by timelapse was about 10-29% less than the clone sizes when these plates were grown in the regular incubator. Thus, we assumed that added stress from the growth conditions in our incucyte and from repeated imaging was significantly affecting these cells and altering their growth dynamics.

As a result of these challenges, we were unable to reliably calculate the transition rates between the states in question. We acknowledge the importance of this data and regret that these experimental difficulties have prevented its inclusion in our current study.

Another approach to approximating transition rates, however, is based upon transcriptional state. In our scRNA-seq analysis, we identified the transcriptional signatures of the highly proliferative and less proliferative clones (Fig. 4E). We then asked whether clones could cross these transcriptional clusters as this could represent a switch between states. We found cells within a specific state and evidence of interconversion. While most clones remained in a single state (547 highly proliferative, 790 less proliferative), we identified around 566 clones exhibiting a mixed state suggestive of transition (Reviewer Figure 7A,B).

Further investigation comparing gene expression between transitioning and non-transitioning cells within lineages revealed no entirely new genes (Reviewer Figure 7C,D). Instead, we observed a pattern of gradual upregulation in transitioning cells of genes upregulated in less proliferative clones, suggesting the absence of a distinct intermediate state. This analysis effectively quantified the extent of phenotype interconversion within lineages, providing a clearer picture of the dynamics at play within these cell populations.

3) The authors note that the proliferative state is heritable though a "limited number of cell division" but don't show what this limit is. It would be good to include data that support this claim and shows that the correlation decays with time.

Thank you for your insightful suggestion about exploring the heritability of the proliferative state in relation to the number of cell divisions and how this correlation might change over time.

In our study, as shown in Figure 2B, we initially restricted the growth of clones to 4 cell divisions before we split them. During this limited time frame, we observed that the growth potential was partially heritable among cells originating from the same parent.

Building on this observation, we extended our focus to longer time scales to determine the maximum limit of this heritability. In our 96-well plate experiments, detailed in Figure S1B, we allowed the cells to grow for 14 days, which equates to more than 10 cell divisions. Over this extended period, we found that the heritability of growth potential

completely diminishes, leading to clones to grow similar to the original bulk distribution of highly proliferative and less proliferative cells.

We also tried to extend this framework to earlier time points. Here, we took proliferative clones after 2-11 days and tried replating them in 96-well plates to quantify what percentage of the progeny would proliferate. However, we found that when trypsinizing small clones after 2-5 days, we often lost the cells during the replating process. This made it impossible to quantify the heritability using this framework. In the future, it could be conceivable to use the barcoding experimental design in Fig. 3 to further investigate this question.

Given these limitations, we have updated the text of our manuscript to more accurately reflect the scope of our findings about heritability and the known bounds on this time frame.

4) The observation that density impacts growth kinetics is important but very well known. It would be good if the authors could clarify how the information they are providing is new compared to what is known in the field.

We appreciate your comment on the well-established understanding of how cell density affects growth kinetics, as highlighted in Figure 3C of our study. In response, we have revised the text, providing relevant citations [15–19], and reframed the context of this experiment to underscore its contribution to the field.

Our aim in conducting this experiment was to probe deeper into the interaction between cell density and clonal growth differences. Although it is widely recognized that increased cell density can enhance growth rates, our focus was on understanding whether such conditions would lead to uniformity in clone sizes or if variations would persist. Specifically, we considered two scenarios: one where increased density uniformly boosts growth rates from a base level, resulting in a wide range of clone sizes, but with overall enhanced growth, and another where increased density leads to homogenized growth rates across all cells, culminating in a more uniform distribution of clone sizes.

The results presented in Figure 3C align with the latter outcome, indicating that under increased density conditions, most clones grow to similar sizes over a specified period, effectively diminishing clonal disparities. This finding adds a new dimension to the existing knowledge by illustrating how varying density conditions can influence the uniformity of clonal growth, a nuance that extends beyond the general understanding of density's impact on cell growth.

We found it interesting that the cell intrinsic phenotype could be overcome by changing density. This finding suggests that density is more important in determining fate than the intrinsic cellular programs. Our study thereby contributes to a more nuanced understanding of cell growth dynamics in relation to clonal diversity and density.

5) My biggest concern is with regards to expansion signature. The authors define a transcriptional state that is associated with expanded clones. This state is associated with Pi3K and WNT. It would be good to corroborate that this signature doesn't disappear with different cell cycle regression methods as well is to show how general it is. For instance, is this specific to esophageal epithelium or a more general cellular trait.

We appreciate your concerns regarding the robustness and generalizability of the transcriptional state associated with expanded clones, particularly in relation to Pi3K and WNT pathways. In response to multiple reviewers' valid concerns regarding the in vivo applicability of our identified signature, we have carefully revised our manuscript.

We have taken steps to ensure the robustness of the identified transcriptional state and have also fine-tuned our assertions to better reflect the core results of our research. We now place a stronger emphasis on relating the less proliferative state to previously known tissue cell types. While honing the relevance of our highly proliferative clones within the context of the healthy human esophagus.

To address the question of the robustness and reliability of our highly proliferative signature, we have regressed out the cell cycle and added a detailed explanation in the methods ("*scRNA-seq cell cycle regression* ") of how this was accomplished. Briefly, we used specific gene sets for the S-phase and G2/M-phase, as identified in previous studies [2]. Using Seurat's CellCycleScoring function, we scored each cell for these phases. Subsequently, we used Seurat's ScaleData function to adjust for cell cycle-related variations in gene expression. This allowed us to ensure that our gene expression analysis was reflective of biological changes, not just cell cycle effects.

We observe distinct clusters representing our highly proliferative and less proliferative clonal states (Fig. S5A and also shown below, left). As a sanity check, Fig. S5C (shown below, right) shows the number of cells per clone as determined by the lineage barcodes and it is clear that the cell cycle regression truly separates out the highly proliferative and less proliferative clones.

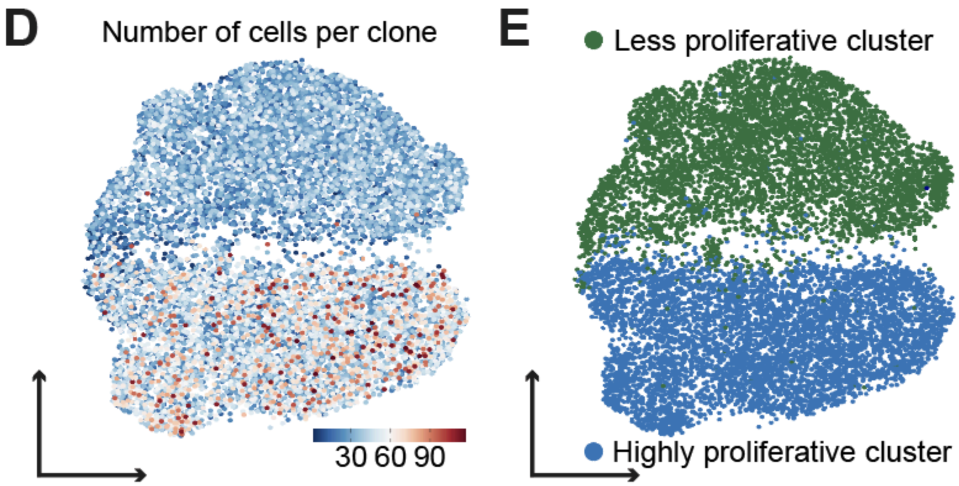

Fig. 4D,E

We have also detailed in the methods how the highly proliferative signature was determined through differential gene expression analysis, comparing cells from highly proliferative clones to those from less proliferative clones. We used a fold change cutoff and adjusted p-values and identified 9 genes that constitute the highly proliferative signature.

Once again, thank you for your invaluable feedback. We believe that the revisions guided by your comments will greatly enhance the quality and clarity of our manuscript.

References

1. Korsunsky I, Millard N, Fan J, Slowikowski K, Zhang F, Wei K, et al. Fast, sensitive and accurate integration of single-cell data with Harmony. Nat Methods. 2019;16: 1289–1296.

2. Tirosh I, Izar B, Prakadan SM, Wadsworth MH 2nd, Treacy D, Trombetta JJ, et al. Dissecting the multicellular ecosystem of metastatic melanoma by single-cell RNA-seq. Science. 2016;352: 189–196.

3. McGinn J, Hallou A, Han S, Krizic K, Ulyanchenko S, Iglesias-Bartolome R, et al. A biomechanical switch regulates the transition towards homeostasis in oesophageal epithelium. Nat Cell Biol. 2021;23: 511–525.

4. Zhang Y, Bailey D, Yang P, Kim E, Que J. The development and stem cells of the esophagus. Development. 2021;148. doi:10.1242/dev.193839

5. Kabir MF, Karami AL, Cruz-Acuña R, Klochkova A, Saxena R, Mu A, et al. Single cell transcriptomic analysis reveals cellular diversity of murine esophageal epithelium. Nat Commun. 2022;13: 2167.

6. Rochman M, Wen T, Kotliar M, Dexheimer PJ, Ben-Baruch Morgenstern N, Caldwell JM, et al. Single-cell RNA-Seq of human esophageal epithelium in homeostasis and allergic inflammation. JCI Insight. 2022;7. doi:10.1172/jci.insight.159093

7. Harada H, Nakagawa H, Oyama K, Takaoka M, Andl CD, Jacobmeier B, et al. Telomerase Induces Immortalization of Human Esophageal Keratinocytes Without p16INK4a Inactivation1. Mol Cancer Res. 2003;1: 729–738.

8. Andl CD, Le Bras GF, Loomans H, Kim AS, Zhou L, Zhang Y, et al. Association of TGFβ signaling with the maintenance of a quiescent stem cell niche in human oral mucosa. Histochem Cell Biol. 2016;146: 539–555.

9. Kotelnikov VM, Coon JS, Taylor S, Hutchinson J, Panje W, Caldareill DD, et al. Proliferation of epithelia of noninvolved mucosa in patients with head and neck cancer. Head Neck. 1996;18: 522–528.

10. Zheng SC, Stein-O'Brien G, Augustin JJ, Slosberg J, Carosso GA, Winer B, et al. Universal prediction of cell-cycle position using transfer learning. Genome Biol. 2022;23: 41.

11. Gier RA, Reyes Hueros RA, Rong J, DeMarshall M, Karakasheva TA, Muir AB, et al. Clonal cell states link Barrett's esophagus and esophageal adenocarcinoma. bioRxiv. 2023. p. 2023.01.26.525564. doi:10.1101/2023.01.26.525564

12. Pan X, Wang J, Guo L, Na F, Du J, Chen X, et al. Identifying a confused cell identity for esophageal squamous cell carcinoma. Signal Transduct Target Ther. 2022;7: 122.

13. Nowicki-Osuch K, Zhuang L, Jammula S, Bleaney CW, Mahbubani KT, Devonshire G, et al. Molecular phenotyping reveals the identity of Barrett's esophagus and its malignant transition. Science. 2021;373: 760–767.

14. Zheng B, Ko K-P, Fang X, Wang X, Zhang J, Jun S, et al. A new murine esophageal organoid culture method and organoid-based model of esophageal squamous cell neoplasia. iScience. 2021;24: 103440.

15. Niimi S, Hayakawa T, Tanaka A. Effect of cell density on induction of growth hormone receptors by dexamethasone in primary cultured rat hepatocytes. Biochem Biophys Res Commun. 1991;174: 928–933.

16. Richardson TP, Trinkaus-Randall V, Nugent MA. Regulation of basic fibroblast growth factor binding and activity by cell density and heparan sulfate. J Biol Chem. 1999;274: 13534–13540.

17. Casella GT, Wieser R, Bunge RP, Margitich IS, Katz J, Olson L, et al. Density dependent regulation of human Schwann cell proliferation. Glia. 2000;30: 165–177.

18. Amsler K. Role of cell density/cell-cell contact, and growth state in expression of differentiated properties by the LLC-PK1 cell. J Cell Physiol. 1994;159: 331–339.

19. Aoki K, Kumagai Y, Sakurai A, Komatsu N, Fujita Y, Shionyu C, et al. Stochastic ERK activation induced by noise and cell-to-cell propagation regulates cell density-dependent proliferation. Mol Cell. 2013;52: 529–540.
