# Supplementary material for: Non-genetic differences underlie variability in proliferation among esophageal epithelial clones
Source: PLoS Comput Biol. 2024 Oct 28;20(10):e1012360. doi: 10.1371/journal.pcbi.1012360 (PMC11573201; doi:10.1371/journal.pcbi.1012360)
Supplement: Attachment — Submitted filename: PLOS Comp Bio Response to Reviewers Final.pdf [file pcbi.1012360.s013.pdf]

Dear Dr. Shaffer,

Thank you very much for submitting your manuscript "Non-genetic differences underlie variability in proliferation among esophageal epithelial clones" for consideration at PLOS Computational Biology. As with all papers reviewed by the journal, your manuscript was reviewed by members of the editorial board and by several independent reviewers. The reviewers appreciated the attention to an important topic. Based on the reviews, we are likely to accept this manuscript for publication, providing that you modify the manuscript according to the review recommendations.

In particular, one relevant point raised by the reviewers concerns the number of replicates. The authors should specify that the scRNA-seq was performed once. For all other experiments at least 2 biological replicates are needed. This is especially true for Fig S5, 5C, S6B, S6C, S6D and S6F which apparently only have technical replicates. The authors should also address the two issues raised by reviewer 1 (regarding the definition of the threshold based on live cell imaging data, and capture effectiveness of small clones). Comments 4 and 10 of reviewer 2 should also be addressed.

Please prepare and submit your revised manuscript within 30 days. If you anticipate any delay, please let us know the expected resubmission date by replying to this email.

When you are ready to resubmit, please upload the following:
[1] A letter containing a detailed list of your responses to all review comments, and a description of the changes you have made in the manuscript. Please note while forming your response, if your article is accepted, you may have the opportunity to make the peer review history publicly available. The record will include editor decision letters (with reviews) and your responses to reviewer comments. If eligible, we will contact you to opt in or out
[2] Two versions of the revised manuscript: one with either highlights or tracked changes denoting where the text has been changed; the other a clean version (uploaded as the manuscript file).

> We appreciate the thoughtful feedback provided by the reviewers. We have addressed their comments and made further revisions to our manuscript to improve its clarity.
>
> We added biological replicates to all experiments that previously lacked them, incorporating data we had already collected and new data. These replicates have been added to the figures in their appropriate sections. We have also updated the methods section for "Clonal Isolation and Replating" by renaming it “Single-cell isolation and subcloning” for clarity and adding a more detailed description of the methods. Additionally, we created a new supplemental figure, now referred to as Figure S2, to quantify the extent of bimodality in the clone size data.
>
> We have removed one experiment from the manuscript since our initial submissions. This experiment quantified PI3K activity using a FOXO1 reporter. Recently, our research lab had an equipment failure and lost some of our cell lines stored in liquid nitrogen. Unfortunately, this EPC2 FOXO1 reporter was lost at this time. After emails with the editors, we have modified the text accordingly to emphasize the claims that can be made from our data. Importantly, we show that inhibiting PI3K activity leads to smaller clones, but we do not experimentally confirm that the highly proliferative clones have higher PI3K activity (which is only suggested by scRNA-seq).

Point-by-point response to reviews for PLOS Comp Bio

Responses text is in blue

Reviewer #1:

Just some additional explanations:

1) The authors gave a clear explanation how the 10% threshold is selected based on experimental observations. However, it is only mentioned that "A small fraction of clones expanded to fill entire wells, while the majority exhibited reduced growth or stopped growing entirely", or in the 6-well plates/live cell imaging condition, the authors mentioned the presence of two types of clones. Yet, the authors did not give enough evidence in how the clone classification were binarized. It is possible that the clone size is a range of values, not necessarily following a bimodal distribution. Especially in the live cell imaging figures (Fig. S2 E and F), the change from the green to the blue lines can be perceived as gradual. The authors need further statistical tests/ plots to supports the assumption of a bimodal distribution of clone sizes.

> We thank the reviewer for their insightful feedback. Importantly, we do not claim that the clone sizes themselves are bimodal in nature with the highly proliferative producing one distribution and the less proliferative producing another. Rather, the difference between these two types of clones lies in whether the majority of the cells proliferate exponentially. Within the less proliferative clones, we do see a range of clone sizes, with some stopping at 2-4 cells, while others do grow larger. The growth models in Fig. 2 and Fig. S3 better captures the observed growth dynamics in these populations.
>
> To further address the reviewer's comment, we have added a supplemental figure showing the distribution of clone sizes across time with a new statistical analysis to quantify the likelihood of bimodality and to rule out unimodality in the data, see Fig. S2 shown below and incorporated into the manuscript. For this analysis, we fit two Gaussian distributions to the clone size data from each time point. We then calculated a bimodality coefficient from these two distributions to quantify the extent to which the distribution is bimodal. These coefficients range from 0 to 1 and values greater than 5/9 suggest bimodality of the data. In Fig. S2 shown below, the bimodality coefficient is shown for different time points and the 5/9 cutoff is given by the gray dashed line in the figure. At all time points, the bimodality coefficient is greater than this cutoff with days 4 and 5 showing the highest bimodality coefficient. This statistical quantification further supports our classification of these different clones into two distinct groups with different growth patterns.
>
> We do note, however, that these two distributions do not perfectly align with the highly versus less proliferative cells at each time point as discussed further above. Among the less proliferative clones, we have less cells actively cycling (as we observe in our single-cell data), but these clones do grow to a range of different sizes. The key difference is that they do not exhibit unlimited growth. In this analysis, we quantify the degree to which this data is not described by a unimodal distribution. The growth differences in these clones are best characterized by our modeling approaches in Fig. 2 and Fig. S3.
>
> Additionally, as the reviewer points out, we observed smaller clone sizes in our live cell imaging experiments. Compared to the same time point in standard conditions, the clones from the live cell imaging experiments were approximately 10-29% smaller. We believe this reduction in size is from the stress of the live-cell imaging, including continuous exposure to light and the temperature fluctuations.

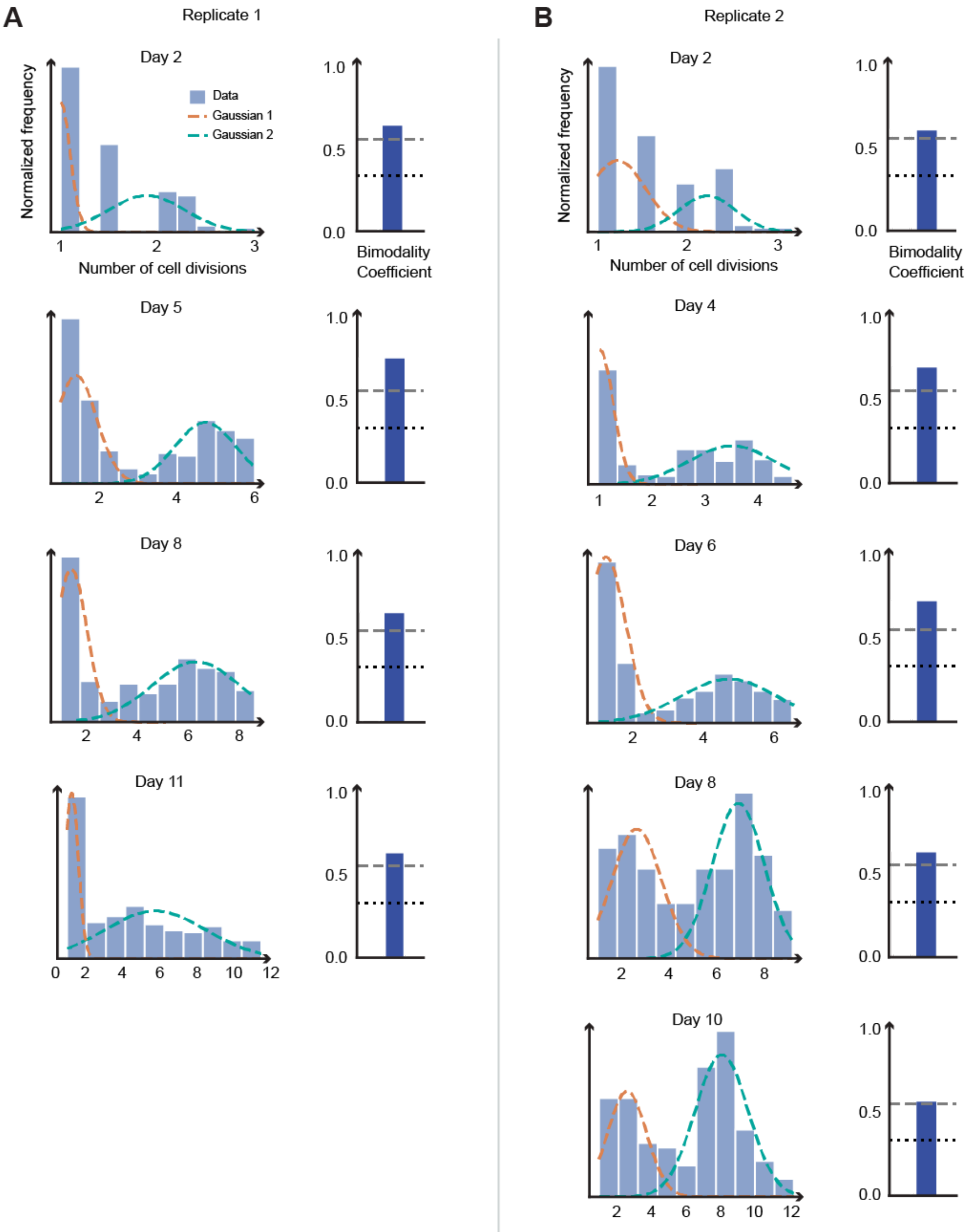

Fig. S2. Bimodality quantification analysis on clone size data across time and replicates. (A-B) Histograms showing the number of cell divisions for each clone based on the number of cells observed. Each set column corresponds to a separate biological replicate. Histograms are fitted with two Gaussians using a Gaussian Mixture Model (GMM). Each histogram is paired with a bar plot on the right, depicting the corresponding Bimodality Coefficient. The dashed gray lines indicate the 5/9 threshold (suggestive of a likely bimodal distribution) and the dashed black lines indicate the 1/3 threshold (suggestive of a unimodal distribution).

2) Though the authors argued that small clones can be more effectively recovered when using 6-well plates, it is not obvious to the reviewer why the size of the plates reduce RNA-seq capture effectiveness of small clones. Thus, the authors should provide further explanation or data to support the statement.

While using 6-well plates, we found that collecting cells is significantly easier and more reliable compared to 96-well plates. We believe the key advantage is the reduced number of pipetting steps required when working with larger wells. Each pipetting action carries a risk of cell loss, either through adherence to the pipette walls or well surfaces. In the 6-well plates, fewer pipetting steps minimize this risk, thereby improving the recovery of small clones. Additionally, collecting cells for single-cell RNA sequencing (scRNA-seq) is highly time-sensitive. The time required to trypsinize and collect cells from a 6-well plate is considerably shorter than that from a 96-well plate. This reduced collection time is critical in minimizing the exposure of cells to external stressors, which can otherwise alter gene expression profiles and affect the accuracy of the RNA-seq data.

Using 6-well plates not only simplifies and speeds up the cell collection process but also facilitates the scaling of experiments. The ease of handling and quicker processing times make it more feasible to maintain consistency and reproducibility across multiple experiments.

We appreciate the reviewers constructive comments and hope these clarifications effectively address their concerns.

Reviewer #2:

Major comments:

4- What is Supplementary Figure 6A trying to show? Is it that both phenotypes express equivalent levels of the reporter? If not… is it the translocation of the reporter? If the latter, I cannot see any difference in nuclear vs cytoplasmic staining. Please include a figure where the difference in nuclear vs cytoplasmic staining for the reporter is clearly visible. Also, is the data analysis here done using confocal microscopy? Or regular fluorescence microscopy? By the description in M&M is just fluorescence microscopy with all the quantification caveats associated to it (e.g fluorescence bleeding, quality of lenses filters, etc). I strongly suggest the authors to re-run the analysis using confocal microscopy.

We have decided to remove the PI3K reporter experiments from the manuscript. Our lab recently had an equipment failure leading to loss of many of our cell lines stored in liquid nitrogen. The EPC2 cell line tagged with the PI3K/FOXO1 receptor was not able to be recovered for experiments including additional replicates and confocal microscopy. Moreover, given that these experiments contributed minimally to the message of the manuscript, we decided to remove this section from the manuscript. We still have data showing PI3K pathways targets in the single-cell RNA-sequencing data and the effects of a PI3K inhibitor on clone size.

10- In the section Clonal isolation and replating in the M&M section there is not description about how this is done. Is FACS based? CellCellector? Is it based on GFP expression? I suggest the authors to include all the data related to single cell sorting in the form of supplementary figures for every time they show a lentiviral transduction, specify gating conditions and percentages obtained. Also display final enrichment after sorting. This data should already be available to the authors.

We recognize the reviewers' confusion here. We have updated this section and renamed it as “Single-cell clone isolation and subcloning”. We have added a detailed description of our workflow in which we count cells and dilute to a concentration such that we can plate at 0.5 cells per well in a 96-well plate. This allows us to isolate single cells. After 8 hours for the cells to adhere, we use

microscopy to determine which wells have only one cell and exclude all other wells from our experiments. We further describe how we allow 14 days for these to grow and then subclone from one well at a time allowing us to isolate another round of single-cells that are all derived from the same clone. We do not use FACS in this workflow.
In our barcoding experiments, we do use flow cytometry, but the purpose for this is not to isolate single cells, rather to isolate the cells that have been successfully transduced with a barcode as our barcode library had a GFP. These methods are described in "*Fluorescence-Activated Cell Sorting*". We have lab notebook entries from these FACS plots, but do not have the original data (See plot below).

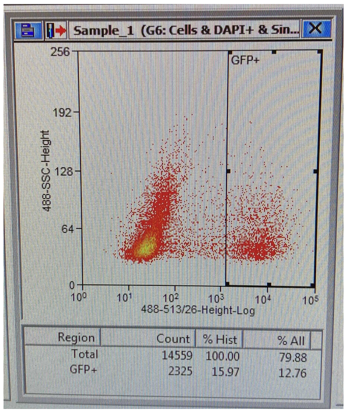

Minor comments:

1- The "Lentiviral transfection" title in the M&M section does not work as it does not reflect what is actually done.

The methods have been revised and "Lentiviral transfection" has been replaced for "Lentivirus production".

2- Some of the "transcriptional state" are still there. Please replace for, as suggested by the authors, gene expression or something alike.

The manuscript has been combed through for the "transcriptional state" phrase and has been replaced with the appropriate terminology.

We thank the reviewer for their insightful comments and for helping clarify the methods.

Reviewer #3:

All issues were addressed
